# The Fragile X Messenger Ribonucleoprotein 1 Regulates the Morphology and Maturation of Human and Rat Oligodendrocytes

**DOI:** 10.1002/glia.24680

**Published:** 2025-02-10

**Authors:** Vidya Ramesh, Eleni Tsoukala, Ioanna Kougianou, Zrinko Kozic, Karen Burr, Biju Viswanath, David Hampton, David Story, Bharath Kumar Reddy, Rakhi Pal, Owen Dando, Peter C. Kind, Sumantra Chattarji, Bhuvaneish T. Selvaraj, Siddharthan Chandran, Lida Zoupi

**Affiliations:** ^1^ Centre for Clinical Brain Sciences University of Edinburgh Edinburgh UK; ^2^ UK Dementia Research Institute University of Edinburgh Edinburgh UK; ^3^ Anne Rowling Regenerative Neurology Clinic (ARRNC) University of Edinburgh Edinburgh UK; ^4^ Euan MacDonald Centre for MND Research University of Edinburgh Edinburgh UK; ^5^ Simons Initiative for the Developing Brain University of Edinburgh Edinburgh UK; ^6^ Centre for Brain Development and Repair Institute for Stem Cell Biology and Regenerative Medicine Bangalore India; ^7^ Centre for Discovery Brain Sciences University of Edinburgh Edinburgh UK; ^8^ National Institute of Mental Health and Neurosciences Bangalore India; ^9^ Centre for High Impact Neuroscience and Translational Applications Kolkata India; ^10^ Patrick Wild Centre University of Edinburgh Edinburgh UK; ^11^ National Centre for Biological Sciences Tata Institute of Fundamental Research Bangalore India

**Keywords:** fragile X syndrome, myelin, neurodevelopmental disorder, oligodendrocytes

## Abstract

The Fragile X Messenger Ribonucleoprotein (FMRP) is an RNA binding protein that regulates the translation of multiple mRNAs and is expressed by neurons and glia in the mammalian brain. Loss of FMRP leads to fragile X syndrome (FXS), a common inherited form of intellectual disability and autism. While most research has been focusing on the neuronal contribution to FXS pathophysiology, the role of glia, particularly oligodendrocytes, is largely unknown. FXS individuals are characterized by white matter changes, which imply impairments in oligodendrocyte differentiation and myelination. We hypothesized that FMRP regulates oligodendrocyte maturation and myelination during postnatal development. Using a combination of human pluripotent stem cell—derived oligodendrocytes and an *Fmr1* knockout rat model, we studied the role of FMRP on mammalian oligodendrocyte development. We found that the loss of FMRP leads to shared defects in oligodendrocyte morphology in both rat and human systems in vitro, which persist in the presence of FMRP‐expressing axons in chimeric engraftment models. Our findings point to species‐conserved, cell‐autonomous defects during oligodendrocyte maturation in FXS.

## Main Points

- Loss of FMRP impairs human and rat oligodendrocyte (OL) morphology *in vitro*.
- FMRP‐deficient OLs show impaired maturation when engrafted in hypomyelinated mice.
- Rat Fmr1 knockout OLs show a delay in myelin sheath formation *in vivo*.

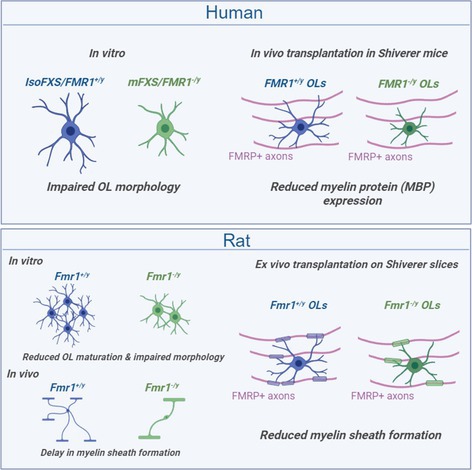

**Abbreviations**
DAPI: 4′,6‐diamidino‐2‐phenylindole
DMEM: Dulbecco's modified eagle medium
FGF: fibroblast growth factor
FMRP: fragile X messenger ribonucleoprotein
FXS: fragile X syndrome
hESC: human embryonic pluripotent stem cell
hiPSC: human induced pluripotent stem cell
hOPC: human oligodendrocyte progenitor cell
hPSC: human pluripotent stem cell
IGF: insulin growth factor
MEM: minimal essential medium
MFOL: myelin forming oligodendrocyte
MNTB: medial nucleus of the trapezoid body
MOL: mature oligodendrocyte
mPFC: medial prefrontal cortex
NFOL: Newly formed oligodendrocyte
OL: oligodendrocyte
OPC: oligodendrocyte precursor cell
PDGF: platelet‐derived growth factor
PFA: paraformaldehyde
PTFE: polytetrafluoroethylene
VLMC: Vascular and leptomeningeal cell

## 1 Introduction

Oligodendrocytes are central nervous system glia that ensheath axons with myelin. Myelin is a lipid rich membrane that insulates and segregates the axons into functional domains enabling fast and energy‐efficient signal transmission (Huxley and Stampfli 1949; Eshed‐Eisenbach, Brophy, and Peles 2023; Nave and Werner 2014). Each oligodendrocyte targets and myelinates multiple axonal segments, which belong to different neuronal subtypes, and generates myelin sheaths that vary in length and in thickness (Pfeiffer, Warrington, and Bansal 1993; Almeida et al. 2011; Tomassy et al. 2014; Zonouzi et al. 2019; Call and Bergles 2021). As such, the formation of each myelin sheath needs to be both spatially and temporally regulated to be able to adapt to the network's requirements. This spatial and temporal resolution is achieved through local mRNA translation that depends on the function of multiple RNA binding proteins (Wake, Lee, and Fields 2011; Herbert et al. 2017; Meservey, Topkar, and Fu 2021; Yergert et al. 2021; Thakurela et al. 2016). Recent studies show that hundreds of mRNAs are localized in the myelin sheaths, with *Fmr1* being identified as one of the highly enriched mRNAs in the nascent myelin sheaths in zebrafish (Yergert et al. 2021; Doll, Yergert, and Appel 2020). Mutations in the *FMR1* gene lead to one of the most common single‐gene causes of intellectual disability and autism, fragile X syndrome (FXS) (Richter and Zhao 2021).

FXS individuals experience a variety of symptoms including restrictive and repetitive behaviors, anxiety, hyperactivity, social avoidance, language impairments, and high incidence of seizures (Yu and Berry‐Kravis 2014). FXS is caused by a CGG repeat expansion in the 5′UTR region of the *FMR1* gene, leading to transcriptional silencing and loss of fragile X messenger ribonucleoprotein (FMRP) from both neurons and glia in the brain (Verkerk et al. 1991; Richter, Bassell, and Klann 2015). FMRP is an RNA binding protein with multiple mRNA targets acting primarily as a translational repressor (Richter, Bassell, and Klann 2015; Ashley Jr. et al. 1993; Siomi et al. 1993; Darnell and Klann 2013). As such, a common feature among FXS preclinical models and human FXS‐derived cells is the dysregulation of protein homeostasis that contributes to the development of several neuronal abnormalities (Richter, Bassell, and Klann 2015; Louros et al. 2023; Seo et al. 2022; Osterweil et al. 2010; Utami et al. 2020; Raj et al. 2021). Despite the important advances behind the molecular and cellular mechanisms underlying neuronal dysfunction, proposed neuron‐focused treatments have not produced robust results in large clinical trials so far (Berry‐Kravis et al. 2022, 2017, 2016; Youssef et al. 2018; Ligsay et al. 2017), while the contribution of glia in FXS pathophysiology remains largely understudied.

FMRP is expressed throughout the oligodendrocyte lineage including mature oligodendrocytes in humans and in rodents (Wang et al. 2004; Giampetruzzi, Carson, and Barbarese 2013; Gholizadeh, Halder, and Hampson 2015; Marques et al. 2016; Zhang et al. 2014). Furthermore, longitudinal imaging studies including in infants and young children with FXS have identified alterations in white matter structures and changes in fiber densities when compared to typically developed individuals (Barnea‐Goraly et al. 2003; Swanson et al. 2018; Hoeft et al. 2010). Studies in *Fmr1* knockout mice showed significant delays during postnatal myelination in the cerebellum and hypomyelination of the auditory brainstem in the adult (Pacey et al. 2013; Lucas et al. 2021). Furthermore, recent evidence from zebrafish showed that *Fmr1* is required for oligodendrocyte differentiation during larval stages and formation of myelin sheaths of appropriate length (Doll, Yergert, and Appel 2020; Doll, Scott, and Appel 2021). Despite the emerging evidence, it is yet unclear how FMRP regulates mammalian oligodendrocyte function.

We hypothesize that FMRP regulates oligodendrocyte maturation and myelination during postnatal development. To test our hypothesis, we used a combination of human pluripotent stem cell (hPSC)—derived oligodendrocytes (*FMR1* knockout and FXS individual‐derived) and *Fmr1* knockout rats to assess the impact of FMRP loss on mammalian oligodendrocyte development. We found that the loss of FMRP leads to cell‐autonomous defects in oligodendrocyte maturation and morphology in both rat and human systems in vitro. Furthermore, using ex vivo and in vivo chimeric engraftment experiments, we show that these cell‐autonomous defects significantly impair their myelination potential ex vivo and morphology in vivo. Collectively, our data identify oligodendrocyte‐specific dysregulations due to the loss of FMRP in both human and rat oligodendrocytes, providing evidence that is of potential translational relevance to human FXS.

## 2 Methods

### 2.1 Animals

Rats and mice were housed and used in accordance with the guidelines established by the Animal Care (Scientific Procedures) Act 1986 and under the authority of Home Office Licenses in the UK and in accordance with the guidelines of the CPCSEA, Government of India and approved by the Institutional Animal Ethics Committees of the National Centre for Biological Sciences and the Institute for Stem Cell and Regenerative Medicine in India. The rats were male Long‐Evans Hooded wild type and *Fmr1* ^*em1*/*PWC*^ rats, hereafter referred to as *Fmr1* ^+/*y*^ and *Fmr1* ^−/*y*^ respectively (Asiminas et al. 2019). For the in vitro *and* in vivo analysis, *Fmr1* ^+/*y*^ and *Fmr1* ^−/*y*^ rats were used. Shiverer mice (*Mbp* ^shi/shi^ –C3Fe.SWV‐MbpShi/J;001428) and *Rag1* mice (NOD.129S7(B6)‐*Rag1* ^*tm1Mom*^/J) were purchased from Jackson laboratories. *Mbp* ^*Shi*/+^; *Rag1* ^−/−^ males were bred with *Mbp* ^*Shi*/+^; *Rag1* ^*−*/*−*^ females to obtain *Mbp* ^*shi*/*shi*^; *Rag1* ^−/−^ male/female pups used for human OPC transplantation experiments. *Mbp* ^*shi*/*shi*^ mouse pups of both sexes were used for the organotypic slice culture experiments. Both mice and rats were bred in‐house and kept on a 12 h/12 h light/dark cycle with ad libitum access to food and water. Following weaning on postnatal day 21 (P21), rats and mice were group housed in mixed‐genotype cages of 2–5 animals per cage. Animals transplanted with the same genotype of human oligodendrocyte precursor cells (hOPCs) were housed together.

### 2.2 Rat OPC Isolation

Cortices from neonatal rat pups (P0‐P3) were isolated following dissection. The cortical tissue was digested using a papain solution, containing MEM (32,360,026, Life Technologies), N‐Acetyl‐L‐cysteine (24 mg/mL, A8199, Sigma‐Aldrich), DNase type IV (40 μg/mL, D5025, Sigma‐Aldrich), papain (30 U/mL, Worthington Biochemical, LS003126), and incubated for 1–1.5 h at 37°C and 5% CO_2_. Digested cortices were then washed with OPC growth medium containing DMEM (41966029, Life Technologies), 10% fetal bovine serum (10270106, Life Technologies), 1% penicillin–streptomycin solution (15140122, Life Technologies), and triturated to single cell suspensions. Cell suspensions were transferred into Poly‐D‐lysine (1 μg/mL, P6407, Sigma‐Aldrich) coated 75 cm^2^ flasks. The mixed glia cultures were kept in growth medium with frequent renewal every 48 h for 10 to 14 days at 37°C and 5% CO_2_. OPC isolation was performed after sequential shaking for 1 h at 220 rpm and at 37°C for microglial removal, followed by further 16 h at 37°C. The next day the OPC‐enriched supernatants were incubated for a further 25 min in 10 cm petri dishes at 37°C and pooled according to genotype. 70,000 OPCs were seeded in 18 mm, Poly‐D‐lysine coated glass coverslips in proliferation medium containing: DMEM (high glucose) + 0.5% fetal bovine serum + 1% penicillin–streptomycin solution, 1% 100x SATO, 1% ITS liquid media supplement (I3146, Sigma‐Aldrich), with freshly added PDGF‐AA (10 ng/mL, 100‐13A, PeproTech) and FGF (10 ng/mL, 100‐18B, PeproTech) until day two in vitro. SATO medium contained 10 mg/mL BSA fraction V (A‐4919, Sigma‐Aldrich), 6 μg/mL Progesterone (P‐8783, Sigma‐Aldrich), 1.61 mg/mL Putrescine (P‐5780, Sigma‐Aldrich), 40 μg/mL L‐Thyroxine T4 (T‐1775, Sigma‐Aldrich) and 40 μg/mL Tri‐iodothyroxine (T‐6397, Sigma‐Aldrich). After 2 days, the proliferation growth factors were replaced by T3 (0.4 μg/mL) and T4 (0.4 μg/mL) to promote cell differentiation until day six in vitro. After 6 days in vitro, our cultures also contained approximately 7% of GFAP expressing cells (*Fmr1* ^+/*y*^: 7.073% ± 0.9836% GFAP+ cells/total cells, *n* = 3 experiments, *Fmr1* ^*−*/*y*^: 7.776% ± 2.808% GFAP+ cells/total cells, *n* = 3 experiments; *p* = 0.7149), while the presence of IBA1 expressing microglia was rare (*Fmr1* ^+/*y*^: 0.1566% ± 0.1167% IBA1+ cells/total cells, *n* = 3 experiments, *Fmr1* ^−/*y*^: 0.1946% ± 0.1293% IBA1+ cells/total cells, *n* = 3 experiments; *p* = 0.7247).

### 2.3 Ex Vivo *Mbp* ^*shi/shi*^ Mouse Cortical Organotypic Slices

P0–P3 *Mbp* ^*shi*/*shi*^ mouse pups were decapitated and their brains were dissected in cold Hibernate‐A medium (A1247501, Thermo Scientific) on ice and the meninges were removed. The brains were immediately mounted on the vibratome and 250‐300 μm coronal cortical slices were collected in ice cold Hibernate‐A medium. The slices were immediately transferred onto Millicell cell culture inserts (30 mm, hydrophilic PTFE, 0.4 μm, PICM0RG50, Merck‐Millipore) using a bent spatula and in warm slice medium containing 50% MEM (32360026, Life Technologies) with 25% Earle's Balanced Salt Solution (24010043, Life Technologies), 25% heat‐inactivated horse serum (26050088, Thermo Scientific), 1% Glutamax supplement (35050061, Thermo Scientific), 1% penicillin–streptomycin, 0.5% Amphotericin B (5290018, Thermo Scientific), and 6.5 mg/mL glucose (G8769, Sigma‐Aldrich). The slices were kept in serum‐containing medium for approximately 5 days and gradually switched to serum‐free medium containing DMEM/F12 (11320033, Thermo Scientific), 1% B‐27 supplement (17504044, Thermo Scientific), 0.5% N2 supplement (17502048, Thermo Scientific), 1% Glutamax supplement, 1% penicillin–streptomycin and 0.5% Amphotericin B before the addition of rat OPCs (Bin et al. 2012). Slices were kept at 37°C and 5% CO_2_ throughout the experiment. The medium was changed every 2 days. After approximately 10 days in culture, 100,000 rat *Fmr1* ^+/*y*^ or *Fmr1* ^−/*y*^ cells were seeded on each cortical slice and cultured for two further weeks before fixation.

### 2.4 Generation of Human Glial Spheres and Oligodendrocytes

The human embryonic stem cell line (male, Shef4, Table S1), referred to as *FMR1* ^+/*y*^ was obtained from the UK Stem Cell Bank (Aflatoonian et al. 2009). Gene‐editing was performed on this line to generate the Shef4 *FMR1* knockout hESC line referred to as *FMR1* ^−/*y*^, as described previously (D'Souza et al. 2018). GM07072 (fragile X syndrome male; Table S1) fibroblasts were obtained from the Coriell Institute for Medical Research under their consent and privacy guidelines as described (http://catalog.coriell.org/). Induced human pluripotent stem cells were generated from this line, referred to as mutant FXS or mFXS, at Cedar‐Sinai Medical Center (Los Angeles, CA) using standard protocols as previously described (Sharma et al. 2023; Das Sharma et al. 2020). The mFXS line carrying the CGG repeat expansion was gene‐corrected using previously published protocols (Ran et al. 2013; Selvaraj et al. 2018) to generate an isogenic control hiPSC line referred to as IsoFXS (Table S1). All experiments were performed after obtaining statutory institutional ethical clearances under full Ethical/Institutional Review Board approval at the University of Edinburgh, UK and Institutional Committee for Stem Cell Research (IC‐SCR), InStem, Bangalore, India. The characterization and validation of all human pluripotent stem cell lines used in this study were performed as described previously (Sharma et al. 2023; Das Sharma et al. 2020; Mehta et al. 2021). Generation of oligodendrocyte cultures from hPSCs was performed according to previous published protocols (Livesey et al. 2016). Embryo bodies were generated by cell lifting with Dispase 1 mg/mL (17105‐041, Life Technologies) and Collagenase 2 mg/mL (17104‐019, Life Technologies) and cultured for 7 days, with dual‐SMAD inhibition, and in chemically defined media (CDM) that contained 50% Iscove's modified Dulbecco's medium (12440053, Thermo Scientific), 50% F12 medium (31765027, Thermo Scientific), BSA (5 mg/mL, 05470, Sigma‐Aldrich), 1% chemically defined Lipid concentrate (11905031, Gibco), monothioglycerol (450 μM, M6145, Sigma‐Aldrich), human insulin (7 mg/mL, 11376497001, Roche), transferrin (15 mg/mL, 10652202001, Roche), 1% penicillin/streptomycin (15070063, Gibco), supplemented with N‐acetyl‐L‐cysteine (1 mM, A7250, Sigma), Activin inhibitor SB43152 (10 μM, 1614, Tocris), and LDN193189 (2 μM, SML0559, Merck Millipore). Generated neurospheres were then caudalized by the addition of retinoic acid (1 μM, R2625, Sigma‐Aldrich) for a further 7 days. Spheres were ventralized with the addition of the sonic hedgehog agonist purmorphamine (1 μM, 483367‐10‐8, Calbiochem) for 7 days in Advanced DMEM/F12 (Invitrogen) containing: 1% N‐2 supplement (17502048, Invitrogen), 1% B27 supplement (17504044, Invitrogen), 1% penicillin/streptomycin, 0.5% Glutamax supplement (35050061, Invitrogen), and 5 μg/mL Heparin (Sigma‐Aldrich). Spheres were further expanded in the presence of basic fibroblast growth factor (FGF)‐2 (10 ng/mL, 45033, PeproTech) for 7 days, following which neural differentiation was induced by FGF2 withdrawal for a further 2 weeks. The resultant glial spheres were further expanded for 2–4 weeks in oligodendrocyte proliferation media containing FGF2 (10 ng/mL), PDGF‐AA (20 ng/mL, 100‐13A, PeproTech), purmorphamine (1 μM), SAG (1 μM, 364590‐63‐6, Calbiochem), IGF‐1 (10 ng/mL, 100‐11, PeproTech), T3 (60 ng/mL, Sigma‐Aldrich), and 1 × ITS (41,400,045, Gibco) before initiating oligodendrocyte differentiation. Terminal differentiation of oligodendrocytes was achieved in oligodendrocyte differentiation media by mitogen withdrawal for 7 days (except for T3 and IGF‐1) following single‐cell dissociation using Papain (20 units/mL) dissociation kit (Worthington Biochemical, LK003150) and plating on Matrigel (SLS, 354230; 1 in 100 dilution), Fibronectin (20 μg/mL, F2006, Sigma‐Aldrich), Laminin (10 μg/mL, L2020, Sigma‐Aldrich) coated coverslips at a density of 10,000 cells per 0.3 cm^2^ until fixation.

To analyze the numbers of OPCs and proliferating cells in the glial spheres, 7–9‐week‐old spheres were plated on 13 mm coverslips coated with Matrigel, Laminin, and Fibronectin as mentioned above. 3–4 spheres were plated per coverslip and cultured for 2 days until fixation.

### 2.5 Human OPC Transplantation in *Mbp* ^*shi/shi*^ Mice Pups

*Mbp* ^*shi*/*shi*^;*Rag1* ^−/−^ homozygous P0‐P1 pups were anesthetized with isoflurane and maintained on a heat mat during transplantation and during recovery. *FMR1* ^+/*y*^ or *FMR1* ^−/*y*^ glial spheres were dissociated at 7–9 weeks of age and injected into rostral and caudal positions within each cerebral hemisphere (70,000 cells per μL, four injections in total per animal) using a Hamilton syringe (24530, Sigma‐Aldrich) and 30G needle. Injections were directed towards the corpus callosum and surrounding cortical gray matter. All pups in one litter were injected with the same genotype of OPCs. At 11–12 weeks of age, only the double homozygous animals were perfused under terminal anesthesia using first ice‐cold PBS, followed by fixation with 4% PFA (252549, Sigma‐Aldrich) and brains collected for histology.

### 2.6 Fluorescence Activated Cell Sorting

Seven‐day old oligodendrocyte cultures of *FMR1* ^+/*y*^ and *FMR1* ^−/*y*^ were dissociated using Accutase (07920, STEMCELL Technologies) and washed twice with oligodendrocyte differentiation medium. The cell pellets were resuspended with Fc block (130‐059‐901, Miltenyi Biotec) for 20 min, followed by incubation with O4 antibody (mouse monoclonal, R and D systems, MAB1326, 1:1000) for 45 min. Following antibody incubation, the pellet was washed twice with PBS and incubated with secondary antibody (mouse monoclonal, Thermo Scientific, A21042, 1:1000) for 30 min. The pellets were washed twice with PBS and resuspended at the concentration of 1 million cells/ml in oligodendrocyte differentiation medium. A sample with only secondary antibody and no primary antibody was used to assess gating for O4+ cells for FACS. Samples were analyzed and isolated on BD FACSAria III machine. O4+ enriched populations were sorted into oligodendrocyte differentiation medium and centrifuged for 10 min at full speed at 4°C. The pellet was resuspended in 100 μL of RLT buffer from the RNeasy micro kit (Qiagen) for RNA extraction and qRT‐PCR.

### 2.7 cDNA Synthesis and qRT‐PCR

#### 2.7.1 Rat

RNA was extracted from *Fmr1* ^+/*y*^ and *Fmr1* ^−/*y*^ day 6 oligodendrocyte cultures using the High Pure RNA Isolation Kit (11828665001, Roche). Total RNA was reverse transcribed into cDNA using the RevertAid First Strand cDNA Synthesis Kit (K1621, Thermo Scientific) according to the manufacturer's instructions. qRT‐PCRs were run on a Stratagene Mx3000P QPCR System (Agilent Technologies) using Power SYBR Green PCR Master Mix (4367659, Thermo Scientific) with 6 ng of cDNA per reaction and with the following programme: 10 min at 95°C, 40 cycles of 30 s at 95°C, 40 s at 60°C and 30 s at 72°C, followed by a cycle of 1 min at 95°C and 30 s at 55°C up to 95°C for 30 s. Fold change was calculated using the Delta Delta Ct method (Livak and Schmittgen 2001) and normalized to rat *Gapdh* expression. Primer sequences used were: Rat *Gapdh* (Forward 5′‐3′: AGTTGGTGGTGCAGGATGC and Reverse 5′‐3′: AGAAGGCTGGGGCTCACC). Rat *Mtor* (Forward 5′‐3′: TGCCTTCACAGATACCCAGT and Reverse 5′‐3′: GCTGCTCGGATGATGTCAAG). Rat *Akt* (Forward 5′‐3′: CCCTTCCTTACAGCCCTCAA and Reverse 5′‐3′: CGTTCTTCTCGGAGTGCAAG). Rat *Pak1* (Forward 5′‐3′: ACGCCCAGAACACACAAAAT and Reverse 5′‐3′: CCACACTCACTATGCTCCGTA). Rat *Srf* (Forward 5′‐3′: AACCAAGGACACACTGAAGC and Reverse 5′‐3′: TGCCTGTACTCTTGAGCACA). Rat *Daam2* (Froward 5′‐3′: CGCACTCGTTTCCAGACTTT and Reverse 5′‐3′: TCTCGTGTTGCCGGAGTTTA). Rat *Tppp* (Forward 5′‐3′: AGCAAGATCAAAGGGAAGTCC and Reverse 5′‐3′: CTTTCGTGACGCCAGAGATG). Rat *Chd2* (Froward 5΄‐3΄: GGAGCGTCTACCACTGTGTA and Reverse 5′‐3′: GAGAAACTTTCCCTAACCATTGC).

#### 2.7.2 Human

RNA was extracted from 7 to 9‐week‐old glial spheres using the RNeasy mini kit from Qiagen (74104) and from FACS isolated 7‐day‐old *FMR1* ^+/*y*^ and *FMR1* ^−/*y*^ oligodendrocyte cultures using the RNAeasy micro kit from Qiagen (74004). RNA was converted to cDNA using RevertAid First Strand cDNA Synthesis Kit (K1621, Thermo Scientific). qPCR was performed using the DyNAmo ColorFlash SYBR Green qPCR Kit (F416, Thermo Scientific) and CFX96 Touch Real‐time PCR detection system (Bio‐Rad). The following programme was used: 5 min at 95°C, 45 cycles of 30 s at 95°C, 30 s at 60°C, melting curve 65°C to 95°C ith 0.5°C increments for 5 s. Primers used were as follows—human *FMR1* (Forward—TCCAATGGCGCTTTCTACA and Reverse—CATCATGAAATGGAATCTGCCTATC), human *GAPDH* (Forward—TCGGAGTCAACGGATTTGGT and Reverse—TTCCCGTTCTCAGCCTTGAC), human *PAK1* (Forward—GCAGCAAAGATGCTGGAACC and Reverse—CTGCTCTGGCATTCCCGTAA), human *mTOR* (Forward—GAAGCCGCGCGAACCT and Reverse—CTGGTTTCCTCATTCCGGCT), human *RAC1* (Forward—TCCGCAAACAGATGTGTTCTTAAT and Reverse—CGCACCTCAGGATACCACTTT), human *CHD2* (Forward—AACAAGAGAATGCAAGGCCC and Reverse—TCCTAGGCCCATTTCATCAGC), human *AKT1* (Forward—CTGCACAAACGAGGGGAGTA and Reverse—TCACGTTGGTCCACATCCTG). Fold change was calculated using the Delta Delta Ct method (Livak and Schmittgen 2001) and normalized to human *GAPDH* expression.

### 2.8 Immunofluorescence

#### 2.8.1 Rat

Brain tissue from rats in the second postnatal week was harvested and drop‐fixed in 4% PFA overnight at 4°C before transferred to 1× PBS solution. Older rats were perfused with 4% PFA and the brain tissue was harvested and postfixed further with 4% PFA overnight at 4°C before transferred to 1× PBS solution. The brain was sectioned using a Leica VT1000S vibratome. 100 μm‐thick coronal sections were briefly washed in PBS before blocking with 10% normal horse serum (26050088, Thermo Scientific), 0.3% Triton‐X (X100, Sigma‐Aldrich) in 1× PBS for 2 h at room temperature. For oligodendrocyte myelin sheath tracing experiments, sections were incubated in antigen unmasking solution (H‐3300‐250, Vector Laboratories) at 95°C for 20 min prior to blocking. Primary antibodies were diluted in the same blocking solution and sections were incubated for 48 h at 4°C. After the primary antibody incubation, sections were washed in 1× PBS (3 × 1 h each), incubated with Alexa Fluor secondary antibodies (Thermo Scientific, 1:1000) for further 16 h and counterstained with Hoechst 33342 solution (62249, Thermo Scientific) for nuclear visualization. All slides were mounted using Fluoromount‐G mounting medium (0100‐01, SouthernBiotech).

For immunocytochemistry, the glass coverslips were fixed with 4% PFA for 20 min at room temperature and then washed three times with 1× PBS. Following permeabilization for 10 min at room temperature and in 0.1% Triton‐X in 1× PBS, coverslips were blocked for 30 min in 10% normal horse serum in 1× PBS at room temperature. The primary antibodies were diluted in the same blocking solution and coverslips were incubated for 2 h at room temperature. For the O4 staining, the primary antibody was added on live cells for 2 h prior to PFA fixation. Subsequently, the coverslips were washed in 1× PBS, incubated with secondary antibodies for 1.5 h at room temperature and counterstained with Hoechst 33342 solution before mounting on glass slides with Fluoromount‐G mounting medium.

For organotypic slice immunohistochemistry the slices were washed once with 1× PBS before they were fixed with 4% PFA for 1 h at room temperature. The slices were subsequently rinsed in 1× PBS and blocked in 3% heat‐inactivated horse serum, 2% BSA (A7906, Sigma‐Aldrich), and 0.5% Triton X‐100 in 1× PBS for 2 h at room temperature. Following blocking, the slices were incubated for 48 h with primary antibodies diluted in blocking solution at 4°C. The slices were then washed three times with blocking solution and incubated with the appropriate secondary antibodies overnight at 4°C. Finally, the slices were washed in 1× PBS, counterstained with Hoechst 33342 solution and mounted on a glass microscopic slide using Fluoromount‐G mounting medium.

The primary antibodies used were as follows: against MBP (rat monoclonal, MCA409S, Bio‐Rad, 1:300), Neurofilament‐H (chicken polyclonal anti‐NF‐H, 822601, Biolegend, 1:10.000), OLIG2 (rabbit polyclonal, AB9610, Sigma‐Aldrich, 1:100), CNPase (mouse monoclonal, AMAB91072, Sigma‐Aldrich, 1:2000), PDGFRα (goat polyclonal, AF1062‐SP, Novus Biologicals, 1:200), CC1 (mouse monoclonal, OP80 | Anti‐APC (Ab‐7) Mouse mAb (CC1), OP80, EMD Millipore 1:300), O4 (mouse monoclonal, MAB1326, R&D systems 1:1000), Ki67 (rat monoclonal, 14‐5698‐82, Thermo Scientific 1:800).

#### 2.8.2 Human

For transplantation experiments, mice were perfused with 4% PFA, and the brain was postfixed with 4% PFA overnight at 4°C before being transferred to 30% sucrose/PBS solution for cryoprotection. Brains were frozen in OCT freezing media (Leica, 4020108926) and sectioned using a Leica CM1850 cryostat. Sagittal sections of 16 μm thickness were obtained and kept frozen until use. For immunohistochemistry, slides were briefly washed in 1× PBS before permeabilizing with 0.5% Triton‐X in 1× PBS for 15 min followed by blocking with 10% normal goat serum (S1000, Vector labs), 0.1% Triton‐X in 1× PBS for 1 h at room temperature. Primary antibodies were diluted in the same blocking solution and sections were incubated for 16–20 h at 4°C. After the primary antibody incubation, sections were washed in 1× PBS (3 × 15 min each) and incubated with Alexa Fluor secondary antibodies (Thermo Scientific, 1:1000) for 1 h at room temperature. Following 3 × 15 min washes with 1× PBS, the slides counterstained with DAPI (D9542, Sigma‐Aldrich) for nuclear visualization. All slides were mounted using Fluorsave mounting medium (345789, Merck Millipore) and 24 × 50 mm glass coverslips.

For immunocytochemistry, 7‐day‐old oligodendrocyte cultures or 2‐day‐old glial spheres plated on glass coverslips were fixed with 4% PFA for 15 min at room temperature and then washed three times with 1× PBS. Following permeabilization for 10 min at room temperature with 0.2% Triton‐X in 1× PBS, immunocytochemistry was performed in the same way as the transplantation immunostaining. For PDGFRα and O4 staining, the primary antibodies were added on live cells for 2 h prior to PFA fixation.

The primary antibodies used were as follows: SOX10 (Rabbit polyclonal, AB155279, Abcam, 1:1000), OLIG2 (Rabbit polyclonal, AB9610, Millipore, 1:1000), PDGFRα (Rabbit polyclonal, D13C6, Cell Signaling), MBP (Rat monoclonal, AB7349, Abcam, 1:50), O4 (MAB1326, R&D systems), Human Nuclei (Mouse monoclonal, AB1281, Millipore, 1:500), Ki67 (Rabbit polyclonal, AB9260, Merck Millipore, 1:500), CNPAse (mouse monoclonal, AMAb91072, Atlas, 1:1000), and PLP (mouse monoclonal, MAB388, Sigma‐Aldrich, 1:500).

### 2.9 Image Acquisition and Analysis

Imaging was done either using Leica TCS SP8, Leica Thunder Imager, Olympus FV1000 or Zeiss LSM710 confocal microscopes.

#### 2.9.1 Rat Analysis

For cell density quantification, 2–6 *z*‐stacks of 40 μm thickness (for cerebellum 40× objective, 332.8 μm × 332.8 μm; pixel size 162.5 nm × 162.5 nm, step size: 0.5 μm; for MNTB: 40× objective, 290.91 × 290.91 μm; pixel size 284.09 nm × 284.09 nm; step size: 0.5 μm; for mPFC 63× objective, 184.58 μm × 184.58 μm each; pixel size 180.38 nm × 180.38 nm, step size: 0.5 μm) were obtained from layers 2/3 of the medial prefrontal cortex, the deep white matter of the cerebellum and the medial nucleus of the trapezoid body (MNTB) from both hemispheres. Oligodendrocyte lineage cell numbers and total cell numbers were quantified automatically using IMARIS 3D imaging software (RRID:SCR_007370, spots module). From each rat, 2–3 sections were analyzed and the numbers were averaged for each animal. Cell numbers were normalized to the total number of cells or to the total number of oligodendrocyte lineage cells.

The average sheath number and myelin sheath length were quantified as previously shown (Swire et al. 2019). Briefly, using 63× objective, 184.58 μm × 184.58 μm; pixel size 180.38 nm × 180.38 nm, step size: 0.5 μm, we imaged individual CNPase‐positive oligodendrocytes in layer 2/3 of the medial prefrontal cortex within the whole 100 μm vibratome section or in cortical organotypic slices (step size 0.5 μm and 1024 × 1024 resolution). The internodal length was quantified using the Simple Neurite Tracer plugin and the sheath number was subsequently quantified using the cell counter plugin in Fiji image analysis software (Fiji, RRID:SCR_002285). The intermodal lengths were binned, and a percentage of frequency distribution is depicted. Alternatively, the log_10_ of the lengths was calculated and represented as a % percent frequency.

For the in vitro analysis, 3–4 coverslips were imaged per genotype and per experiment. Each coverslip was scanned using 63× objective and 1024 × 1024 resolution. Composite areas consisting of 45–65 tiles of 184.52 μm × 184.52 μm, each were obtained for the cell density quantification in vitro. Cell densities were quantified with IMARIS 3D imaging software (RRID:SCR_007370, spots module) and Fiji image software. For the Sholl analysis, *z*‐stacks of individual oligodendrocytes (20–40 cells/genotype/experiment and four different experiments) were imaged and analyzed using the Sholl analysis plugin in Fiji with 10 μm concentric circle intervals. The total number of intersections relative to the distance of the cell body is shown.

#### 2.9.2 Human Analysis

For the transplant analysis, sections were imaged using 40× objective (1.5× optical zoom for MBP) and 1024 × 1024 resolution (16 μm‐thick sections, step size 0.5 μm). Areas containing human nuclei in the corpus callosum, and fornix were imaged with 2–3 FOV per section. From each mouse, 2–3 sections were analyzed, and the numbers were averaged for each animal. Pixel density analysis was performed using a CellProfiler (RRID:SCR_007358) pipeline wherein the MBP image threshold was set and the MBP area was quantified per MBP cluster. 10–20 MBP clusters were analyzed per animal. For SOX10 analysis, a CellProfiler automated analysis pipeline was used to quantify colocalised SOX10 and human nuclei and total human nuclei per FOV. For PLP and CNPase, sections were imaged using 63× objective (2× optical zoom) and 1024×1024 resolution (16 μm‐thick sections, step size 0.4 μm).

For the in vitro Sholl analysis, 2–3 coverslips were imaged per genotype and per experiment. Each coverslip was scanned using 40× objective, 1.5 zoom and 1024 × 1024 resolution (step size 0.5 μm). For the Sholl analysis, *z*‐stacks of individual oligodendrocytes (5–15 cells/genotype/experiment and 3–4 different experiments) were imaged and analyzed using the Sholl analysis plugin in Fiji software with 2 μm concentric circle intervals. The total number of intersections relative to the distance of the cell body is shown.

For % OLIG2, Ki67, PDGFRα, and SOX10 over total nuclei analysis in the glial spheres, only cells which had migrated out of the spheres after 2DIV were analyzed. Three fields of view from three spheres were imaged per genotype and per experiment using a 20× objective (step size 1 μm, 1024 × 1024 resolution). For %SOX10+/total cells and %MBP+SOX10+/total SOX10+ in the 7‐day‐old oligodendrocyte cultures, 2–3 coverslips per genotype and per experiment were imaged (step size 1 μm, 1024 × 1024 resolution). The marker positive cells and total nuclei (DAPI positive) were counted manually using the cell counter plugin in Fiji. For %SOX10+/total cells, %MBP+SOX10+/total SOX10+ and SOX10+PDGFRα+Ki67+/total SOX10+ nuclei analysis in 7‐day‐old oligodendrocyte cultures, a CellProfiler automated analysis pipeline was used.

### 2.10 Western Blotting

Postnatal day 21 (3rd week) rat frontal cortices were homogenized in ice‐cold RIPA Buffer (89900, Thermo Scientific) and protease inhibitor cocktail (PI87786, Thermo Scientific). The total protein in each sample was quantified using the Pierce BCA Protein Assay Kit (23225, Thermo Scientific). 20 μg protein extracts were analyzed by sodium dodecyl sulfate polyacrylamide gel electrophoresis (4%–15% Mini‐PROTEAN TGX Precast Protein Gels, 4561084DC, Bio‐Rad) and electro‐transferred to 0.45 μm Protran nitrocellulose transfer membrane (QZY‐ge10600008, Amersham). The total loaded protein was detected using the Thermo Scientific Pierce Reversible Protein Stain kit according to the manufacturer's instructions (PI24580, Fisher Scientific) prior to blocking. Following 1 h blocking (5% powdered skimmed milk and 0.1% Tween 20 in 0.1 M PBS), the membrane was incubated overnight with primary antibodies. Samples were incubated with horseradish peroxidase‐coupled secondary antibodies (31470, Thermo Scientific and GENA931, Sigma‐Aldrich) and proteins were visualized with enhanced chemiluminescence using LI‐COR Odyssey M. The following antibodies were used: MBP (rat monoclonal, MCA409S, Bio‐Rad, 1:2000), CNPase (mouse monoclonal, AMAB91072, Sigma‐Aldrich, 1:1000), mouse GAPDH‐HRP loading control (ab9482, Abcam, 1:1000). The intensity of the bands was measured in Fiji image analysis software and normalized to the GAPDH loading control.

### 2.11 In Silico Analysis

We downloaded the single cell sequencing dataset of oligodendrocyte lineage cells from (Marques et al. 2016). Expression data were imported into Seurat (Hao et al. 2021) (R package version 4.3.0), and the thirteen distinct populations of cells in the data set were consolidated into six major classes: OPC (oligodendrocyte precursor cells), COP (differentiation‐committed oligodendrocyte precursors), NFOL (newly formed oligodendrocytes), MFOL (myelin‐forming oligodendrocytes), MOL (mature oligodendrocytes), and VLMC (vascular and leptomeningeal cells); VLMCs were excluded from further analysis. Per‐cell expression values were normalized using Seurat's “LogNormalize” method, and average gene expression calculated per‐cell class. A set of 7347 expressed genes was constructed, after filtering out genes expressed lower than an average of 1 in 100,000 counts per‐cell.

We then intersected these genes with HITS‐CLIP data of FMRP targets in the postnatal (P11–25) mouse brain (Maurin et al. 2018) to form a background universe of 7066 genes which were both expressed in the single‐cell data and analyzed by Maurin et al., and for each oligodendrocyte cell class, ranked genes by their average expression in that class. We performed gene ontology (GO) enrichment analysis of the 2285 FMRP targets (those genes with PEAKS > 0 as identified in Maurin et al.) in our gene universe, in the biological process category, using topGO (Alexa, Rahnenfuhrer, and Lengauer 2006) (R package version 2.52.0), retaining those GO terms enriched with *p* value < 0.01, and with at least 10 FMRP target genes. For each such GO term, we performed a Kolmogorov–Smirnov test on the ranks of the FMRP targets for that term, in the ranked expression list for each cell class, to determine skewedness of ranks (where the more positive the KS‐test statistic, the more skewed the ranks of the FMRP targets are towards the top of the expression ranking list for a cell class).

### 2.12 Statistical Analysis

Statistical analysis was performed using GraphPad Prism 9 or 10 (RRID:SCR_002798). Data were tested for normal distribution using the D'Agostino–Pearson test or the Shapiro–Wilk test. The variance of the data was assessed using the F test for variance. Depending on data distribution parametric or nonparametric tests were used. A difference was considered statistically significant when *p* < 0.05. Data are shown as mean ± sem or mean ± sd. Details of statistical test used, precise *p* values and *n* values for each comparison are detailed in the main text and figure legends. Illustrations created with BioRender.com.

## 3 Results

### 3.1 Impaired Morphology and Decreased Maturation of Rat *Fmr1* ^*−/y*^ Oligodendrocytes In Vitro

The observed white matter defects in FXS children and in animal models point to dysregulations in myelination and oligodendrocyte function early in postnatal development. As FMRP is expressed by oligodendrocytes (Giampetruzzi, Carson, and Barbarese 2013; Marques et al. 2016; Zhang et al. 2014), we first sought to identify its potential targets within the oligodendrocyte lineage. We integrated a single‐cell sequencing dataset of oligodendrocyte lineage cells (Marques et al. 2016) with HITS‐CLIP data of FMRP targets from the postnatal mouse brain (Maurin et al. 2018). FMRP targets were expressed throughout the oligodendrocyte lineage. To explore the oligodendrocyte‐specific processes that are likely to be disrupted by the loss of FMRP, we subsequently performed a GO enrichment analysis on these targets (Table S2). Enriched GO terms included processes involved in synaptic assembly, cytoskeletal and microtubule organization, glucose homeostasis, and regulation of myelination that are known to be implicated in different stages of oligodendrogenesis and differentiation (Funfschilling et al. 2012; Hughes and Appel 2019; Micu et al. 2017; Mozell and McMorris 1991; Saab et al. 2016; Saab, Tzvetanova, and Nave 2013; Lee et al. 2012; Carson et al. 1997; Meyer et al. 2018; Bauer, Richter‐Landsberg, and Ffrench‐Constant 2009; Zuchero et al. 2015). For most GO terms, OPCs showed the highest enrichment in potential mRNA binding targets within the oligodendrocyte lineage (Figure S1).

**FIGURE 1:**
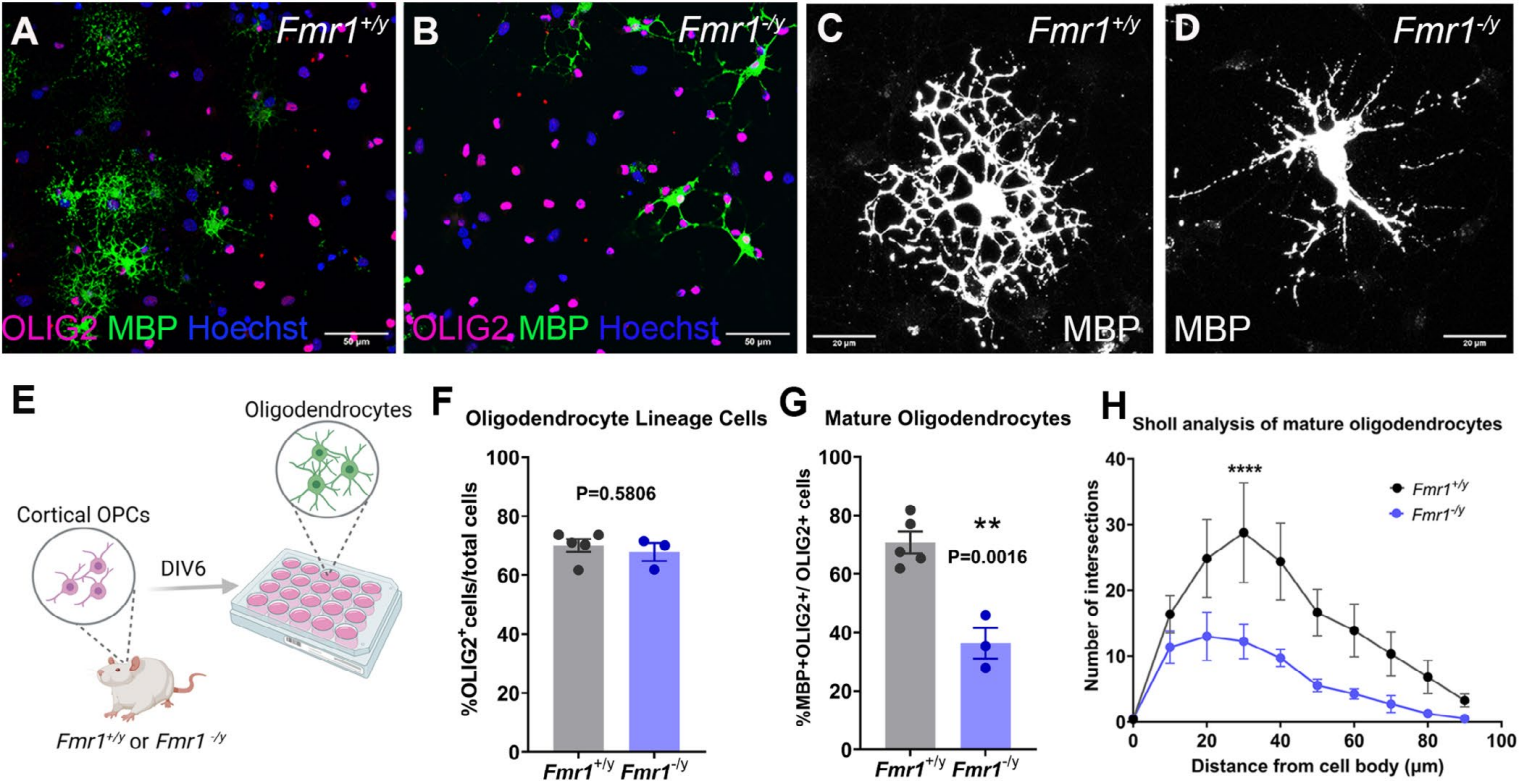
Impaired morphology and maturation of rat *Fmr1* ^*−*/*y*^ oligodendrocytes in vitro. (A, B) Representative images of cortical oligodendrocytes after 6 days in vitro immunostained for OLIG2 (magenta), MBP (green), and counterstained nuclei with Hoechst (blue). (C, D) Representative images of MBP‐expressing mature oligodendrocytes from each genotype after 6 days in vitro. (E) Experimental design. (F) Percent of OLIG2‐expressing cells over the total cell number after 6 days in vitro; data presented as mean ± sem and each circle is an independent experiment (G) Percent of MBP‐expressing cells over the total OLIG2+ cell number after 6 days in vitro; data presented as mean ± sem and each circle is an independent experiment (H) Sholl analysis of oligodendrocyte morphology between genotypes; data presented as mean ± sem of four different experiments. *p* value calculated in (F, G) with two‐tailed, unpaired *t*‐test with Welch's correction and with two‐way ANOVA with Šídák's multiple comparisons test in (H) **** indicates *p* value < 0.0001.

We then sought to determine the oligodendrocyte‐specific effects of FMRP loss at the cellular level in mammalian systems. We isolated OPCs from the cortices of *Fmr1* ^+/*y*^ and *Fmr1* ^*−*/*y*^ rat pups and cultured them in the absence of neurons for 6 days in vitro (Figure 1A,B,E). Following immunocytochemistry for the transcription factor OLIG2 (Figure 1A,B), we first quantified the percentage of OLIG2‐expressing oligodendrocyte lineage cells in our cultures and found no difference between genotypes (*Fmr1* ^+/*y*^: 70.06% ± 4.926% OLIG2+ cells/total cells, *n* = 5 experiments, *Fmr1* ^*−*/*y*^: 67.82% ± 5.237% OLIG2+ cells/total cells, *n* = 3 experiments; *p* = 0.5806) (Figure 1F). Using the cell surface marker platelet‐derived growth factor receptor alpha (PDGFRα) to mark the oligodendrocyte progenitors combined with Ki67 to label proliferating cells (Figure S2A,B), we found no difference in the densities of total OPCs (*Fmr1* ^+/*y*^: 22.45% ± 5.399% PDGFRα+ cells/total cells, *n* = 3 experiments, *Fmr1* ^*−*/*y*^: 26.40% ± 7.503% PDGFRα+ cells/total cells, *n* = 3 experiments; *p* = 0.5049) nor in the densities of proliferating OPCs (*Fmr1* ^+/*y*^: 44.49% ± 15.52% PDGFRα+ Ki67+ cells/PDGFRα+ cells, *n* = 3 experiments, *Fmr1* ^*−*/*y*^: 37.76% ± 6.472% PDGFRα+ Ki67+ cells/PDGFRα+ cells, *n* = 3 experiments; *p* = 0.5430) between genotypes (Figure S2E,F). No significant difference was also observed in the densities of the differentiated oligodendrocytes expressing the oligodendrocyte marker 4 (O4) (Figure S2C,D,G; *Fmr1* ^+/*y*^: 28.55% ± 1.869% O4+ OLIG2+ cells/OLIG2+ cells, *n* = 3 experiments, *Fmr1* ^*−*/*y*^: 37.91% ± 9.693% O4+ OLIG2+ cells/OLIG2+ cells, *n* = 3 experiments; *p* = 0.2337). In contrast, when we measured the percentage of mature and myelin basic protein (MBP)‐expressing oligodendrocytes, we found a significant decrease in the *Fmr1* ^*−*/*y*^ cultures compared with controls (*Fmr1* ^+/*y*^: 70.70% ± 8.392% OLIG2+ MBP+ cells/OLIG2+ cells, *n* = 5 experiments, *Fmr1* ^*−*/*y*^: 36.33% ± 9.051% OLIG2+ MBP+ cells/OLIG2+ cells, *n* = 3 experiments; *p* = 0.0016) (Figure 1G), which suggests an effect during oligodendrocyte maturation and not during the proliferation or differentiation of oligodendrocyte lineage cells in vitro.

We also observed that the morphology of the *Fmr1* ^*−*/*y*^ MBP‐expressing oligodendrocytes was different to that of the *Fmr1* ^+/*y*^ oligodendrocytes (Figure 1C,D). To assess the morphological features of mature oligodendrocytes in our cultures, we performed Sholl analysis on individual MBP‐expressing oligodendrocytes after 6 days in culture (Figure 1H). *Fmr1* ^*−*/*y*^ oligodendrocytes showed simpler morphologies and fewer branching points in vitro than the wild type oligodendrocytes (Genotype effect: *F* = 25.85. DFn = 1, DFd = 50, *p* < 0.0001, *n* = 4 different experiments/genotype).

To identify possible changes in the oligodendrocytic gene expression that could contribute to the morphological and maturation impairments we observe in vitro, we isolated total RNA from day six oligodendrocyte cultures and performed quantitative RT‐PCR. We primarily focused on cytoskeletal candidate genes that were highly enriched in our bioinformatics analysis (Figure S3A). The analysis revealed a 28% reduction in the tubulin polymerization promoting protein (*Tppp*) gene in day 6 *Fmr1* ^*−*/*y*^ oligodendrocyte cultures compared to control (*Fmr1* ^+/*y*^ = 1.006 ± 0.137 and *Fmr1* ^*−*/*y*^ = 0.7155 ± 0.047, *n* = 3 experiments; *p* = 0.0255). These results suggest an oligodendrocyte‐specific morphological defect during late maturation due to the loss of FMRP.

### 3.2 Impaired Morphology of Human *FMR1* ^*−/y*^ and mFXS Oligodendrocytes In Vitro

**FIGURE 2:**
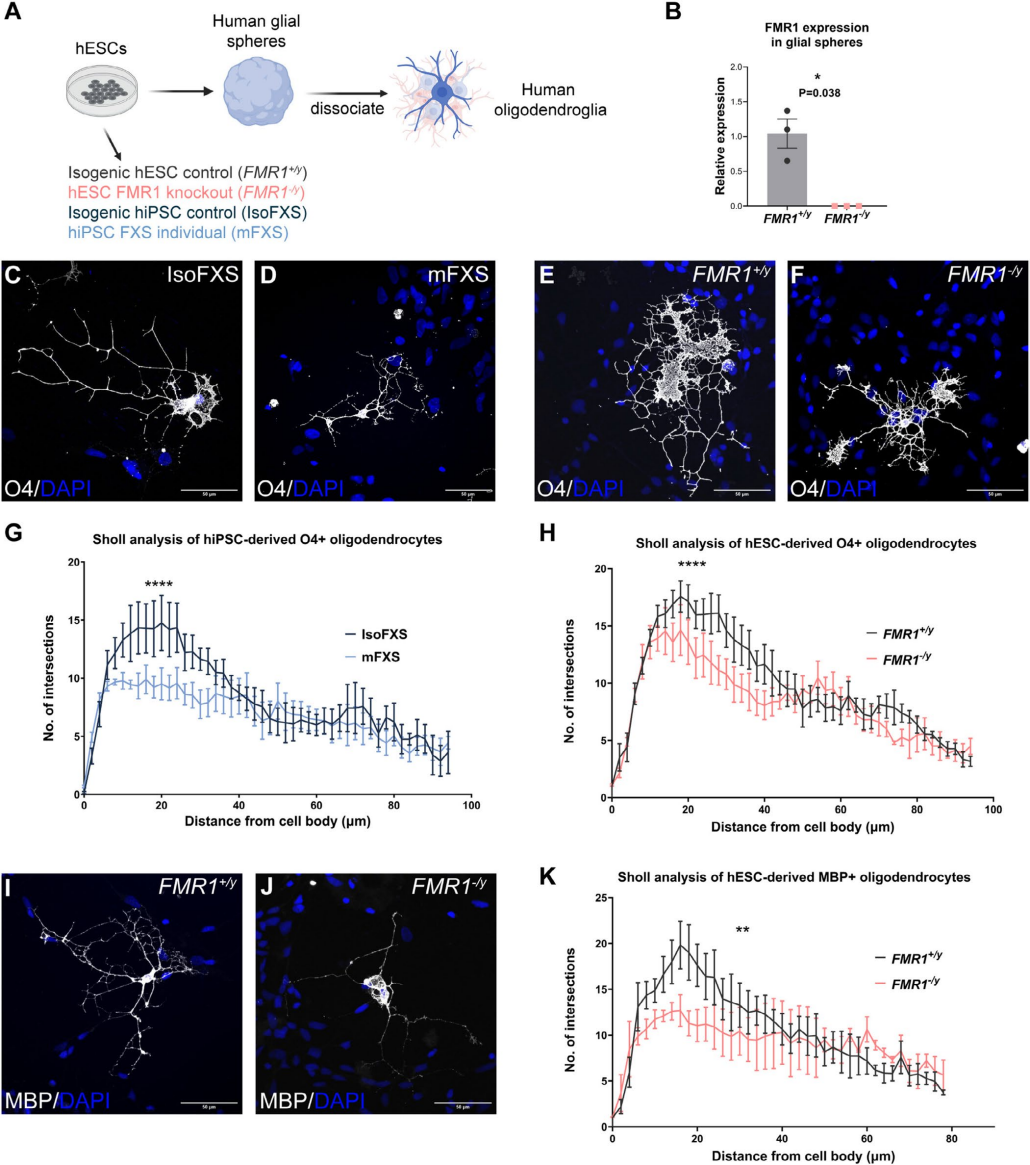
Impaired morphology of human *FMR1* ^*−*/*y*^ and mFXS oligodendrocytes in vitro. (A) Schematic showing generation of human mixed glial cultures containing oligodendrocytes (dark blue), OPCs (light blue) and astrocytes (pink). (B) qRT‐PCR for *FMR1* mRNA in 7–9‐week‐old *FMR1* ^+/*y*^ and *FMR1* ^*−*/*y*^ glial spheres; each data point is an independent experiment. (C–F) Representative images of *FMR1* ^+/*y*^, *FMR1* ^*−*/*y*^, IsoFXS, and mFXS human oligodendrocytes after 7 days in vitro immunostained for O4 (white) and counterstained nuclei with DAPI (blue). (G) Sholl analysis of O4+ oligodendrocyte morphology between IsoFXS and mFXS oligodendrocytes. (H) Sholl analysis of O4+ oligodendrocyte morphology between *FMR1* ^+/*y*^ and *FMR1* ^*−*/*y*^ oligodendrocytes. (I, J) Representative images of *FMR1* ^+/*y*^ and *FMR1* ^*−*/*y*^ human oligodendrocytes after 7 days in vitro immunostained for MBP (white) and counterstained nuclei with DAPI (blue). (K) Sholl analysis of MBP+ oligodendrocyte morphology between *FMR1* ^+/*y*^ and *FMR1* ^*−*/*y*^ oligodendrocytes. Data points and error bars represent means and sem, respectively, from 3 to 4 different experiments; *p* value in (G, H) calculated using two‐way ANOVA and in (B) using two‐tailed unpaired *t*‐test with Welch's correction. **** indicates *p* < 0.0001 and ** indicates *p* = 0.0093.

To determine if the cellular defects of rat *Fmr1* ^*−*/*y*^ oligodendrocytes were also conserved in human oligodendrocytes that lacked FMRP, we generated human embryonic stem cell (hESCs) derived oligodendrocytes using a previously published protocol from our group (Livesey et al. 2016). Glial precursor‐enriched spheres were derived from *FMR1* null hESCs (named *FMR1* ^*−*/*y*^) and from an isogenic hESC control (named *FMR1* ^+/*y*^) and differentiated into glial cultures containing oligodendrocytes (Figure 2A). The downregulation of *FMR1*, which has been previously described by our group (Das Sharma et al. 2020), was confirmed by quantitative RT‐PCR in glial spheres (Figure 2B) (*FMR1* ^+/*y*^ = 1.04 ± 0.21 and *FMR1* ^*−*/*y*^ = 0.0001 ± 3.599e‐005, *n* = 3 experiments; *p* = 0.038). First, we investigated if there were any changes in the generation of oligodendrocyte precursors using immunocytochemistry for OLIG2, SOX10 and PDGFRα across genotypes and found no difference (Figure S4B–J) (*FMR1* ^+/*y*^: 51.62% ± 5.51% OLIG2+ cells/total cells, and *FMR1* ^*−*/*y*^: 49.69% ± 5.99% OLIG2+ cells/total cells, *n* = 4 experiments, *p* = 0.82; *FMR1* ^+/*y*^: 13.3% ± 1.36% PDGFRα+ cells/total cells and *FMR1* ^*−*/*y*^: 20.17% ± 7.29% PDGFRα+ cells/total cells, *n* = 3 experiments, *p* = 0.44, *FMR1* ^+/*y*^: 31.87% ± 7.56% SOX10+ cells/total cells, and *FMR1* ^*−*/*y*^: 29.23% ± 4.82% SOX10+ cells/total cells, *n* = 3 experiments, *p* = 0.78). We further analyzed cell proliferation using immunocytochemistry for Ki67, and found no differences across genotypes (Figure S4K–M) (*FMR1* ^+/*y*^: 37.34% ± 0.24% Ki67+ cells/total cells and *FMR1* ^*−*/*y*^: 31.43% ± 1.45% Ki67+ cells/total cells, *n* = 3 experiments, *p* = 0.052). We further analyzed if proliferating OPCs are specifically affected (Figure S4N–P) and found no differences (*FMR1* ^+/*y*^: 9.91% ± 0.62% SOX10+PDGFRα+Ki67+ cells/total cells, and *FMR1* ^*−*/*y*^: 10.39% ± 2.82% SOX10+PDGFRα+Ki67+ cells/total cells, *n* = 3 experiments, *p* = 0.88). Following this, we generated glial cultures containing oligodendrocytes from *FMR1* ^*−*/*y*^ hESCs and *FMR1* ^+/*y*^ hESCs as well as FXS individual‐derived hiPSCs (mFXS) and isogenic hiPSC control (IsoFXS) (Figure 2A and Table S1). This FXS‐derived iPSC line has been previously characterized by our group to show downregulation of the FMRP protein compared to its gene‐edited control (Sharma et al. 2023). Using immunocytochemistry for SOX10 and MBP in 7‐day‐old glial cultures (Figure S5A–F), we tested for possible changes in the proportions of oligodendrocytes and oligodendrocyte lineage cells and observed no difference across genotypes (Figure S5G,H) (*FMR1* ^+/*y*^: 25.18% ± 1.28% MBP+SOX10+ cells/total SOX10+ and *FMR1* ^*−*/*y*^: 23.24% ± 1.03% MBP+SOX10+ cells/total SOX10+, *n* = 3 experiments, *p* = 0.3; *FMR1* ^+/*y*^: 18.7% ± 9.41% SOX10+ cells/total cells and *FMR1* ^*−*/*y*^: 16.5% ± 9.74% SOX10+ cells/total cells, *n* = 3 experiments, *p* = 0.88; IsoFXS: 19% ± 9.96% SOX10+ cells/total cells and mFXS: 35.3% ± 8.88% SOX10+ cells/total cells, *n* = 3 experiments, *p* = 0.29).

Human oligodendrocytes at this stage (7 days in culture) express O4, which allowed us to assess their morphology using Sholl analysis (Figure 2C–F). *FMR1* ^*−*/*y*^ and mFXS oligodendrocytes showed fewer branching points in vitro than the control *FMR1* ^+/*y*^ and IsoFXS oligodendrocytes, respectively (Figure 2G,H) (*FMR1* ^+/*y*^ and *FMR1* ^*−*/*y*^: Genotype effect: *F* = 25.67, DFn = 1, DFd = 260, *p* < 0.0001, *n* = 4 experiments/genotype; IsoFXS and mFXS: Genotype effect: *F* = 24.72, DFn = 1, DFd = 283, *p* < 0.0001, *n* = 4 experiments/genotype), similar to the effect observed in rat *Fmr1* ^*−*/*y*^ oligodendrocytes. Since O4 is expressed by both mature and immature oligodendrocytes, we also assessed the morphology of MBP+ oligodendrocytes and observed similar findings to O4 (Figure 2I–K) (*FMR1* ^+/*y*^ and *FMR1* ^*−*/*y*^: Genotype effect: *F* = 6.9, DFn = 1, DFd = 145, *p* = 0.0093, *n* = 3 experiments/genotype).

As for the rat cultures, we tested for changes in cytoskeletal genes that could contribute to the impaired morphology of *FMR1* ^−/y^ oligodendrocytes. We isolated O4+ oligodendrocytes from 7‐day‐old cultures using fluorescence activated cell sorting (FACS) (Figure S3B) and performed quantitative RT‐PCR. Our analysis revealed a 60% reduction in p21 activated kinase (*PAK1*) gene expression in 7‐day‐old *FMR1* ^*−*/*y*^ oligodendrocyte cultures compared to controls (Figure S3C) (*FMR1* ^+/*y*^ = 1.016 ± 0.13 and *FMR1* ^*−*/*y*^ = 0.38 ± 0.12, *n* = 3 experiments; *p* = 0.027), hinting toward an oligodendrocyte‐specific morphological defect due to the loss of FMRP. These data suggest that there is a shared role for FMRP on oligodendrocyte maturation and morphology across species.

### 3.3 Cortical Myelination Impairment in the Prefrontal Cortex of *Fmr1* ^*−/y*^ Rats In Vivo

Our analysis of human and rat *Fmr1*‐ null oligodendrocytes identified morphological and maturation defects in vitro. We next asked if these impairments are also detected during postnatal development in vivo in *Fmr1* ^*−*/*y*^ rats (Figure S6A). A previous report in *Fmr1* ^*−*/*y*^ mice, detected transient changes in the densities of OPCs in the deep cerebellar white matter at the end of the first postnatal week (Pacey et al. 2013). Our comparison of rat *Fmr1* ^+/*y*^ and *Fmr1* ^*−*/*y*^ cerebella at the second postnatal week (Figure S6E–H) showed no significant difference between genotypes in OPC densities (*Fmr1* ^+/*y*^: 0.1961 ± 0.024 PDGFRα+ cells/Hoechst cells, *n* = 3 rats, *Fmr1* ^*−*/*y*^: 0.2014 ± 0.025 PDGFRα+ cells/Hoechst cells, *n* = 3 rats, *p* = 0.8032), although we detected relatively fewer cells overall (*Fmr1* ^+/*y*^: 568778 ± 68,723 Hoechst cells/mm^3^, *n* = 3 rats, *Fmr1* ^*−*/*y*^: 426930 ± 66,879 Hoechst cells/mm^3^, *n* = 3 rats, *p* = 0.0625) in the *Fmr1* ^*−*/*y*^ white matter (Figure S6G,H). We then tested the sparsely myelinated layers 2/3 of the medial prefrontal cortex (mPFC) in *Fmr1* ^*−*/*y*^ rats when myelination was still ongoing. Dysfunction of the PFC, a region that is involved in emotional behavior and cognitive functions, has been associated with cognitive impairments in both FXS individuals (Wilding, Cornish, and Munir 2002; Munir, Cornish, and Wilding 2000a, 2000b; Bray et al. 2011) and in *Fmr1* ^*−*/*y*^ rodents (Asiminas et al. 2019; Krueger et al. 2011; Kramvis et al. 2013; Meredith et al. 2007). Furthermore, changes in mPFC myelination have been linked to impaired memory and social interactions (Makinodan et al. 2012; Steadman et al. 2020; Shimizu et al. 2023; Pan et al. 2020). To detect changes in oligodendrocyte lineage cell densities during the third postnatal week (early mPFC myelination), we used immunohistochemistry for PDGFRα, APC (CC1, mature oligodendrocytes) and OLIG2 in layers 2/3 of the mPFC (Figure S6B–D). We did not observe any significant differences in the densities of oligodendrocyte progenitors (*Fmr1* ^+/*y*^: 0.6868 ± 0.03370 PDGFRα+ cells/OLIG2+ cells, *n* = 5 rats, *Fmr1* ^*−*/*y*^: 0.6952 ± 0.01823 PDGFRα+ cells/OLIG2+ cells, *n* = 4 rats, *p* = 0.6494) (Figure S6B), of oligodendrocyte lineage cells (*Fmr1* ^+/*y*^: 0.1311 ± 0.02748 OLIG2+ cells/total cells, *n* = 5 rats, *Fmr1* ^*−*/*y*^: 0.1421 ± 0.01342 OLIG2+ cells/total cells, *n* = 4 rats, *p* = 0.4629) (Figure S6C), or of mature oligodendrocytes (*Fmr1* ^+/*y*^: 0.07847 ± 0.01690 CC1+ cells/OLIG2+ cells, *n* = 5 rats, *Fmr1* ^*−*/*y*^: 0.07072 ± 0.01656 CC1+ cells/OLIG2+ cells, *n* = 4 rats, *p* = 0.5124) (Figure S6D) between genotypes. No difference was observed for both OPCs (*Fmr1* ^+/*y*^: 0.03906 ± 0.005609 PDGFRα+ cells/Hoechst cells, *n* = 3 rats, *Fmr1* ^*−*/*y*^: 0.02916 ± 0.01052 PDGFRα+ cells/Hoechst cells, *n* = 3 rats, *p* = 0.2445) and for mature oligodendrocytes (*Fmr1* ^+/*y*^: 0.1222 ± 0.02997 CC1+ cells/Hoechst cells, *n* = 3 rats, *Fmr1* ^*−*/*y*^: 0.1343 ± 0.02089 PDGFRα+ cells/Hoechst cells, *n* = 3 rats, *p* = 0.6010) in the adult medial nucleus of the trapezoid body (MNTB—Figure S6I–L). This result is different to a previous study in adult *Fmr1* ^*−*/*y*^ mice, which reported significant increases in both OPC and mature oligodendrocyte densities compared to controls (Lucas et al. 2021). In contrast to findings in *Fmr1* ^*−*/*y*^ mice, our results indicate that sufficient numbers of oligodendrocyte cell populations are present in the absence of FMRP in the *Fmr1* ^*−*/*y*^ rats.

**FIGURE 3:**
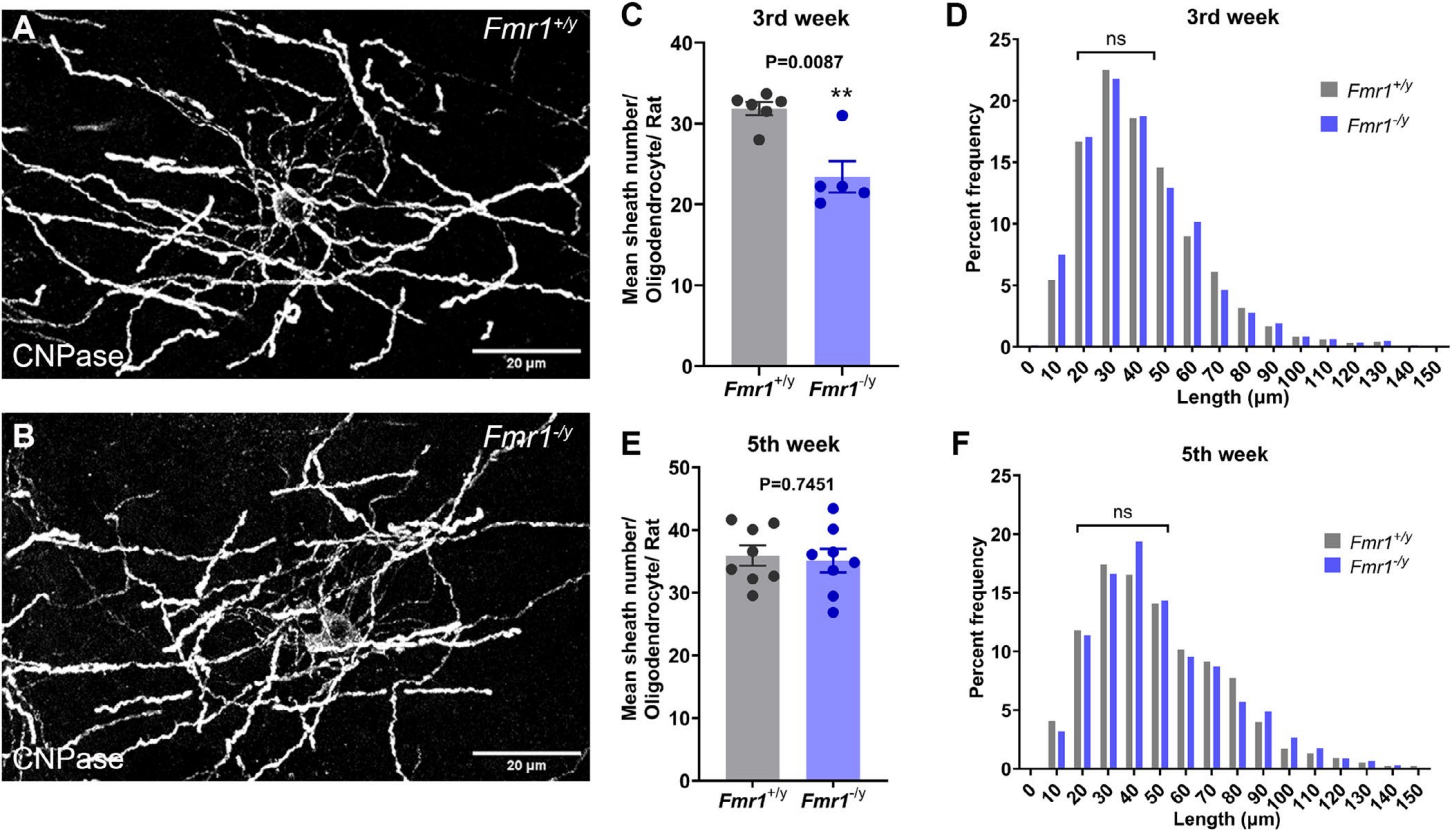
Cortical myelination impairment in the prefrontal cortex of *Fmr1* ^*−*/*y*^ rats. (A, B) Example of myelinating oligodendrocytes at the 3rd postnatal week in the rat mPFC stained for CNPase. (C) Mean myelin sheath number/oligodendrocyte in layers 2/3 of the mPFC during the 3rd postnatal week. (D) Percent frequency of myelin sheath length distribution per genotype at the 3rd postnatal week. (E) Mean number/oligodendrocyte in layers 2/3 of the mPFC during the 5th postnatal week. (F) Percent frequency of myelin sheath length distribution per genotype at the 5th postnatal week. Each data point is a rat. Data presented as mean ± sem. *p* values calculated with two‐tailed Mann–Whitney test for C and two‐tailed, unpaired *t*‐test for the mean sheath number in (E) and mean sheath length in (D and F).

To assess for changes in the properties of myelination in vivo, we next asked if the *Fmr1* ^*−*/*y*^ myelin forming oligodendrocytes generate comparable numbers of myelin sheaths and of similar internodal lengths to the *Fmr1* ^+/*y*^ oligodendrocytes. To address this question, we used a previously established immunohistochemistry method (Swire et al. 2019) that allows the tracing of individual oligodendrocyte cell bodies, their processes and their myelin sheaths using a CNPase antibody (Figure 3A,B). We traced individual oligodendrocytes in layers 2/3 of the mPFC and assessed their morphologies in two different postnatal timepoints (3rd and 5th postnatal week). Our analysis showed that the *Fmr1* ^*−*/*y*^ oligodendrocytes form on average significantly fewer myelin sheaths than the wild types (*Fmr1* ^+/*y*^: 31.85 ± 2.016 sheaths/oligodendrocyte, *n* = 6 rats, *Fmr1* ^*−*/*y*^: 23.41 ± 4.329 sheaths/oligodendrocyte, *n* = 5 rats; *p* = 0.0087), but of comparable lengths (*Fmr1* ^+/*y*^: 41.66 μm ± 5.429 μm,1200 sheaths, *n* = 4 rats, *Fmr1* ^*−*/*y*^: 40.64 μm ± 5.911 μm, 827 sheaths *n* = 3 rats; *p* = 0.8274) during the third postnatal week (Figure 3C,D). However, this difference did not result in a significant reduction in the overall levels of CNPase and MBP proteins in the frontal cortex of the mutant rats when assessed by Western blot (Figure S7A,B). No difference was detected between genotypes at the fifth postnatal week for both the mean myelin sheath number per oligodendrocyte (*Fmr1* ^+/*y*^: 35.93 ± 4.597 sheaths/oligodendrocyte, *n* = 8 rats, *Fmr1* ^*−*/*y*^: 35.10 ± 5.339 sheaths/oligodendrocyte, *n* = 8 rats; *p* = 0.7450) and for the mean internodal length (*Fmr1* ^+/*y*^: 50.80 μm ± 5.313 μm,1280 sheaths, *n* = 3 rats, *Fmr1* ^*−*/*y*^: 50.59 μm ± 3.231 μm, 1354 sheaths, *n* = 3 rats; *p* = 0.9569) (Figure 3E,F). These results show that in addition to the altered oligodendrocyte morphology seen in vitro, *Fmr1* ^*−*/*y*^ oligodendrocytes show early and transient defects in myelin sheath formation in vivo, which are in line with previous findings showing early and transient myelination deficits in the cerebellum of *Fmr1‐*null mice (Pacey et al. 2013).

### 3.4 Impaired Morphology of Human *FMR1* ^*−/y*^ Oligodendrocytes In Vivo in Hypomyelinated Immunosuppressed Mice

**FIGURE 4:**
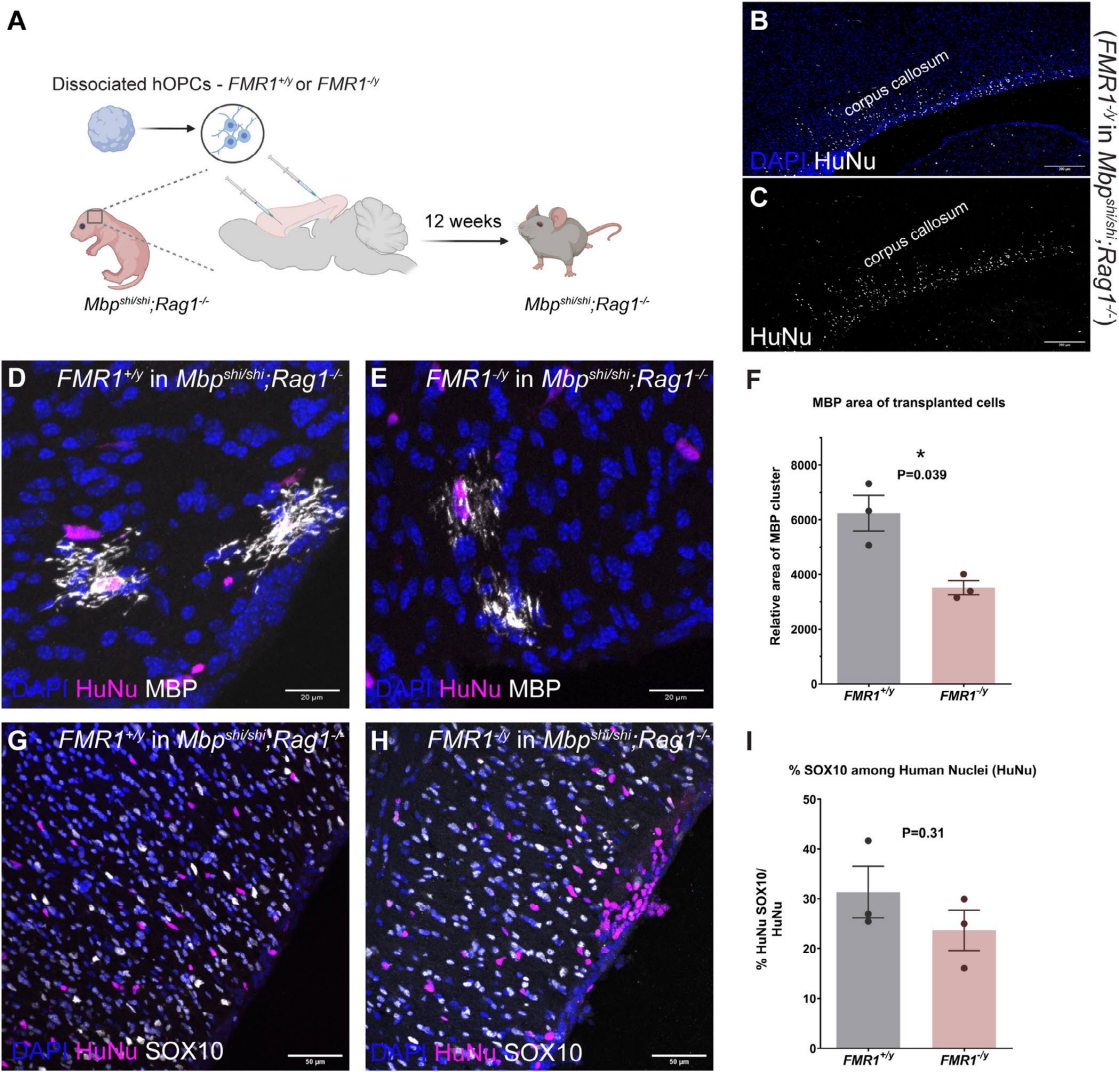
Impaired morphology of transplanted human *FMR1* ^*−*/*y*^ oligodendrocytes in vivo in *MBP* ^*shi*/*shi*^, *Rag1* ^*−*/*−*^ (immunosuppressed shiverer) mice. (A) Schematic of chimeric transplantation paradigm (B, C) Representative image showing spread of human cells in the corpus callosum of *MBP* ^*shi*/*shi*^, *Rag1* ^*−*/*−*^ mice immunostained for Human Nuclei marker (HuNu, white) and counterstained with DAPI (blue). (D, E, G, H) Representative images of transplanted *FMR1* ^+/*y*^ and *FMR1* ^*−*/*y*^ human oligodendrocytes in *MBP* ^*shi*/*shi*^, *Rag1* ^*−*/*−*^ mice at 12 weeks immunostained for MBP (white), Human Nuclei (magenta), SOX10 (white), and counterstained with DAPI (blue) (F) Graph showing relative MBP volume per MBP+ cluster of *FMR1* ^+/*y*^ and *FMR1* ^*−*/*y*^ transplanted oligodendrocytes. (I) Graph showing % SOX10 among Human Nuclei population in *FMR1* ^+/*y*^ and *FMR1* ^*−*/*y*^ transplanted shiverer mice. Each data point is a mouse from a different litter. Error bars indicate sem from three different experiments; *p* values calculated using two‐tailed unpaired *t*‐test with Welch's correction.

To assess if the in vitro morphological defects observed in *FMR1* ^*−*/*y*^ human oligodendrocytes are cell‐autonomous and retained in vivo, we employed an in vivo chimeric model wherein dissociated *FMR1* ^+/*y*^ or *FMR1* ^*−*/*y*^ glial spheres were transplanted into neonatal immunodeficient and hypomyelinated MBP‐deficient mice (*Mbp* ^*shi*/*shi*^; *Rag1* ^*−*/*−*^) that normally express FMRP. Since *Mbp* ^*shi*/*shi*^ mice lack expression of MBP protein (Kirkpatrick et al. 2001; Nave 1994), any MBP expression would derive from the transplanted human oligodendrocytes. P0–P1 neonatal pups were injected with *FMR1* ^+/*y*^ or *FMR1* ^*−*/*y*^ dissociated spheres containing OPCs (four injections of 70,000 cells per injection) into the rostral and caudal neocortex and analyzed at 11–12 weeks post transplantation (Figure 4A). Human nuclei were largely found in the areas surrounding the lateral ventricles (mainly in the corpus callosum) (Figure 4B,C) and in the fornix. To identify the human cells which differentiated into oligodendrocytes, we performed immunohistochemistry for MBP and focused on the clusters of MBP transplanted cells in vivo (Figure 4D–F). Although at this stage, the differentiated human oligodendrocytes had not made many distinct myelin sheaths (Chanoumidou et al. 2021), they co‐expressed MBP along with additional markers such as CNPase and PLP (Figure S8). To assess the transplanted oligodendrocyte morphology, we used pixel density analysis of the MBP+ area per cluster which revealed a reduction in relative area in *FMR1* ^*−*/*y*^ oligodendrocytes compared to *FMR1* ^+/*y*^ oligodendrocytes (*FMR1* ^+/*y*^: 6233 ± 652.6, *FMR1* ^*−*/*y*^: 3520 ± 255.8, *n* = 3 mice, *p* = 0.039). In contrast, the numbers of human SOX10+ oligodendrocyte lineage cells in the *Mbp* ^*shi*/*shi*^; *Rag1* ^*−*/*−*^ brains were comparable between genotypes (*FMR1* ^+/y^: 31.36% ± 5.1784% SOX10+ cells/total Human nuclei+ cells, *FMR1* ^−/y^: 23.67% ± 4.061% SOX10+ cells/total Human nuclei+ cells, *n* = 3 mice, *p* = 0.31) (Figure 4G–I) suggesting that the difference in MBP signal is likely due to impaired differentiation and/or maturation of the *FMR1* ^*−*/*y*^ oligodendrocytes. These data indicate that the impairments in human *FMR1* ^*−*/*y*^ oligodendrocyte maturation persist among mouse axons that express FMRP.

### 3.5 Rat *Fmr1* ^*−/y*^ Oligodendrocytes Form Fewer Myelin Sheaths on *Mbp* ^*shi/shi*^ Axons Ex Vivo

**FIGURE 5:**
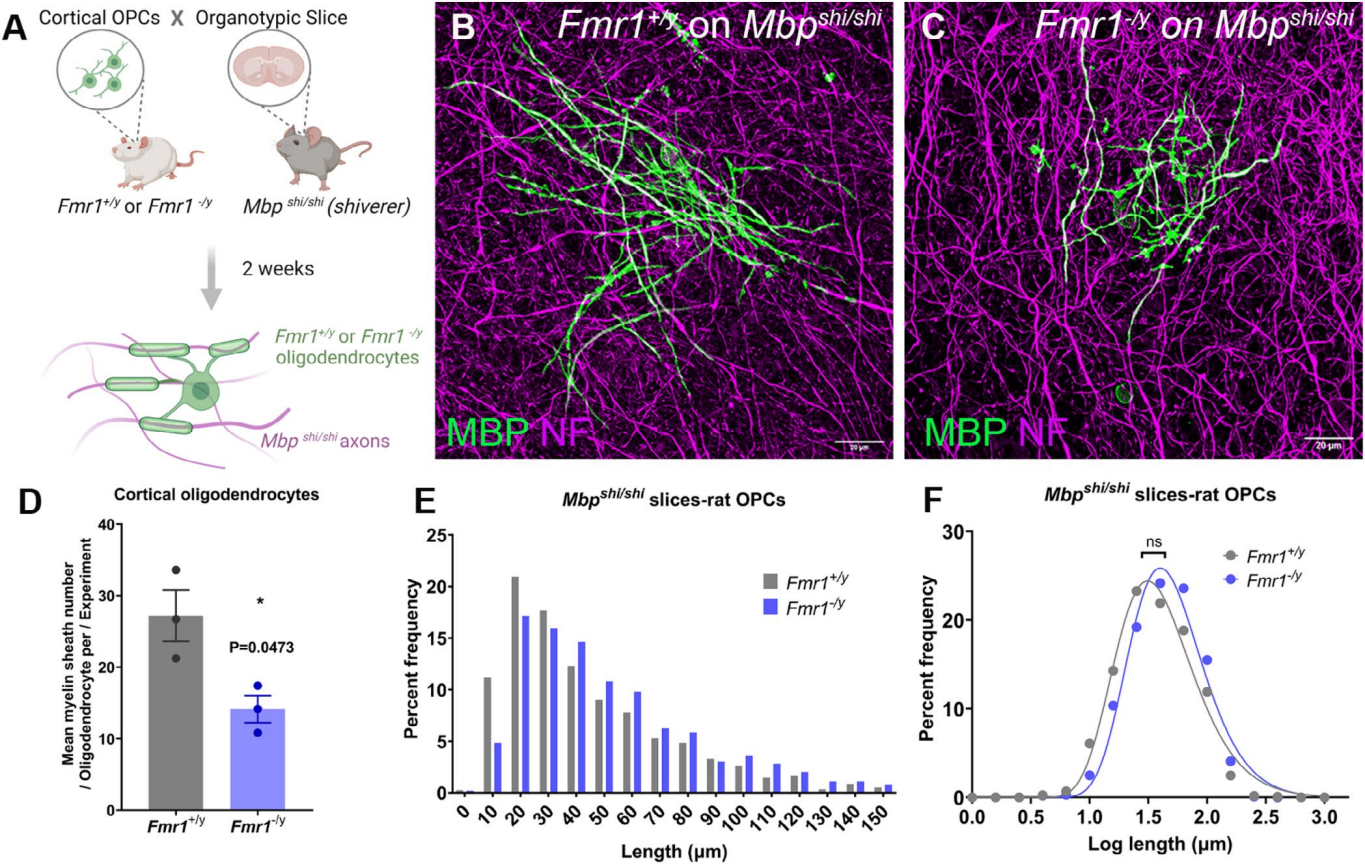
Rat *Fmr1* ^*−*/*y*^ oligodendrocytes form fewer myelin sheaths on *Mbp* ^*shi*/*shi*^ axons ex vivo. (A) Experimental design. (B, C) Representative images of *Fmr1* ^+/*y*^ (B) or *Fmr1* ^*−*/*y*^ (C) rat oligodendrocytes on mouse *Mbp* ^*shi*/*shi*^ (shiverer) axons after 2 weeks in culture, immunostained for neurofilament (NF‐magenta) and MBP (green). (D) Mean myelin sheath number/oligodendrocyte per experiment; data presented as mean ± sem and each circle is an independent experiment. (E) Histogram of sheath lengths formed by *Fmr1* ^+/*y*^ or *Fmr1* ^*−*/*y*^ rat oligodendrocytes on *Mbp* ^*shi*/*shi*^ mouse cortical slices after 2 weeks in culture. (F) Log sheath length plot for *Fmr1* ^+/*y*^ or *Fmr1* ^*−*/*y*^ oligodendrocytes cultured on *Mbp* ^*shi*/*shi*^ cortical slices. Data in (E and F) from three separate experiments per genotype. *p* value calculated with two‐tailed, unpaired *t*‐test with Welch's correction in (D) and two‐tailed unpaired *t*‐test for the mean sheath length and mean sheath log length in (E and F), respectively.

Given that rat *Fmr1* ^*−*/*y*^ oligodendrocytes exhibit morphological and maturation deficits in vitro and myelination impairments in vivo, we sought to determine if these deficits are cell‐autonomous and retained in the presence of FMRP‐expressing axons as in the case of human *FMR1* ^*−*/*y*^ oligodendrocytes. To assess this, we used an ex vivo chimeric co‐culture system in which *Fmr1* ^+/*y*^ or *Fmr1* ^*−*/*y*^ rat oligodendrocyte progenitors were transplanted onto cortical organotypic slices derived from neonatal *Mbp* ^*shi*/*shi*^ mice. *Mbp* ^*shi*/*shi*^ cortices were seeded with 100,000 OPCs and analyzed after 2 weeks of co‐culture to match the early stages of cortical myelination in vivo (Figure 5A). The seeded rat oligodendrocytes were detected both in the corpus callosum and in the cortex using immunohistochemistry for MBP (Figure 5B,C). Individually traced *Fmr1* ^*−*/*y*^ rat oligodendrocytes on *Mbp* ^*shi*/*shi*^ cortices formed significantly fewer myelin sheaths than *Fmr1* ^+/*y*^ oligodendrocytes after 2 weeks in culture (*Fmr1* ^+/*y*^: 27.19 ± 6.189 sheaths/oligodendrocyte, *n* = 3 experiments, *Fmr1* ^*−*/*y*^: 14.14 ± 3.293 sheaths/oligodendrocyte, *n* = 3 experiments; *p* = 0.0473) (Figure 5D) but of comparable internodal lengths (*Fmr1* ^+/*y*^: 42.84 μm ± 20.02 μm, 1168 sheaths, *n* = 4 experiments, *Fmr1* ^*−*/*y*^: 42.77 μm ± 17.79 μm, 1005 sheaths *n* = 4 experiments; *p* = 0.9960) (Figure 5E,F). This indicates that the *Fmr1* ^*−*/*y*^ rat oligodendrocytes form fewer myelin sheaths than wildtypes, on mouse axons that express FMRP.

These data are in accordance with the human in vivo transplantation experiments and together indicate that even in an in vivo environment with multiple *Fmr1*‐expressing cell types, the maturation of *Fmr1*/*FMR1* null oligodendrocytes is impaired, advocating for a cell‐autonomous role for *Fmr1*/*FMR1* in regulating oligodendrocyte maturation and morphology.

## 4 Discussion

FXS is a common monogenic form of inherited intellectual disability and autism with extensive clinical heterogeneity (Verdura et al. 2021) and cellular pathophysiology that urges the need to study the involvement of multiple cell types in the brain, as well as the inclusion of human models to improve therapeutic outcomes. Despite growing evidence of white matter abnormalities in FXS individuals (Barnea‐Goraly et al. 2003; Swanson et al. 2018; Hoeft et al. 2010), the contribution of oligodendrocyte lineage cells in FXS pathophysiology remained largely understudied.

*FMR1* has been previously reported to be expressed in rodent and human OPCs, in pre‐myelinating and in myelinating oligodendrocytes (Wang et al. 2004; Giampetruzzi, Carson, and Barbarese 2013; Marques et al. 2016; Zhang et al. 2014; Pacey et al. 2013; Pacey and Doering 2007). We also confirmed the expression of *FMR1* mRNA in human OPCs and examined its involvement in both human and rat oligodendrocyte development. Analysis of human glial spheres and oligodendrocyte cultures showed that the generation and the proliferation of hOPCs and oligodendrocytes are largely unaffected by the loss of FMRP, indicating that FMRP is not essential for hOPC production and specification. Similarly, the densities of rat oligodendrocyte progenitors and oligodendrocyte lineage cells were unaffected in vitro and in vivo in different brain areas and at different postnatal stages. These findings are in contrast to previous reports in mice that observed a transient reduction in OPCs in the cerebellar white matter early in postnatal development and an increase of both OPCs and mature oligodendrocytes in the adult MNTB (Pacey et al. 2013; Lucas et al. 2021). Furthermore, a study in zebrafish has described an increase in the number of oligodendrocyte lineage cells (SOX10^+^) at early larval stages in *Fmr1* knockout fish (Doll, Scott, and Appel 2021). Changes in the total cell density between genotypes may skew the results for individual cell populations. Our analysis in the cerebellum revealed a lower cell count in the *Fmr1* ^*−*/*y*^ rats and although the difference was not statistically significant, this could potentially explain the discrepancy with mice. However, no other differences were found in any of the brain regions and postnatal stages tested between wild type and mutant rats. This suggests that, if present, any transient differences in density may not have been detected in our analysis, or it may indicate a difference between species.

Our data indicate that although FMRP may not be critical for the proliferation or differentiation of the oligodendrocyte lineage, it is likely important for the maturation of oligodendrocytes and the acquisition of elaborate morphologies that would allow the appropriate myelination of axons in vitro. Our analysis of human and rat *Fmr1*/*FMR1* deficient oligodendrocytes identified an impairment in oligodendrocyte maturation as indicated by the reduced MBP+/OLIG2+ cell densities in the rat mutant cultures and the significantly reduced branching networks of human and rat mutant oligodendrocytes. Oligodendrocyte morphology defects have been described to be regulated by various mechanisms (Osanai et al. 2022; Almeida et al. 2021; Mensch et al. 2015; Ishibashi et al. 2009, 2006), including cell‐intrinsic mechanisms of the cytoskeleton and cell adhesion molecules. In fact, our bioinformatics analysis identified a variety of cytoskeletal regulators such as *Rac1, Pak1, Daam2, Sema6a*, all of which are targeted by FMRP and were shown to affect oligodendrocyte morphology (Thurnherr et al. 2006; Cristobal et al. 2022; Brown et al. 2021; Bernard et al. 2012). Our qRT‐PCR analysis of candidate cytoskeletal genes in *FMR1*/*Fmr1*‐deficient human and rat in vitro cultures revealed that *Pak1* and *Tppp* mRNAs were significantly reduced, respectively. Downregulation of *Pak1* in rat OPC cultures resulted in aberrant oligodendrocyte morphology (Brown et al. 2021). Similarly, mouse *Tppp* knockout oligodendrocyte cultures showed aberrant microtubule branching and myelin sheath impairments (Fu et al. 2019). These results indicate that our observed morphological defects in vitro and possibly in vivo may partly be governed by defects in the oligodendrocyte cytoskeleton, although a more thorough investigation of the underlying mechanisms is required in this case.

Defects in oligodendrocyte maturation in vivo have also been reported in zebrafish that showed reduced *Myrf* (a marker for mature oligodendrocytes) levels in *Fmr1* null larvae (Doll, Scott, and Appel 2021) and in *Fmr1* knockout mice, which displayed abnormal expression of myelin proteins MBP and CNPase (Pacey et al. 2013). In addition, FMRP targets a number of mRNAs that have been identified as oligodendrocyte‐specific regulators of myelination (e.g., synaptic proteins, cell adhesion molecules, and members of the Akt–mToR signaling pathway) that were also highly ranked in the oligodendrocyte lineage cell FMRP target expression list in our in silico analysis (Figure S1, Table S2). In our case, human *FMR1*‐deficient oligodendrocyte cultures showed a trend for reduced *mTOR* expression and a similar decrease of *Akt1* mRNA, alluding to a potential dysregulation of this pathway upon loss of FMRP. Synaptic‐like molecular machinery is often accumulated at the points of axon‐oligodendrocyte contact from the onset of myelination while oligodendrocyte‐specific disruptions in this communication in both mice and in zebrafish impaired axonal myelination (Hughes and Appel 2019; Almeida et al. 2021; Williamson, Lyons, and Almeida 2019; Fang et al. 2022; Korrell et al. 2019; Elazar et al. 2019).

Although we did not observe significant differences in the densities of mature oligodendrocytes in vivo in rats, we did observe a significant reduction in the average number of myelin sheaths they form during the third postnatal week. This defect was not accompanied by a reduction in the average myelin sheath length and was not evident at the fifth postnatal week in the rat, which suggests a myelination delay caused by the reduced formation of myelin sheaths in the medial prefrontal cortex. Nevertheless, both *Fmr1*/*FMR1* knockout rat and human oligodendrocytes retained this morphological impairment when transplanted onto hypomyelinated FMRP‐expressing mouse axons ex vivo and in vivo, respectively. This implies that cell‐autonomous and conserved defects in oligodendrocytes can lead to in vivo delays in myelination during early postnatal development that may affect the establishment and the subsequent formation of neuronal networks. Oligodendrocytes receive inputs from neurons and respond to changes in neuronal activity through a variety of neurotransmitter receptors on their membrane (Karadottir et al. 2008; Lin and Bergles 2004; Etxeberria et al. 2010; De Biase, Nishiyama, and Bergles 2010; Kougioumtzidou et al. 2017; Hamilton et al. 2017; Spitzer et al. 2019, 2016). These dynamic changes are now recognized as a new form of brain plasticity that regulates the performance of neuronal networks and is important for many aspects of cognition such as social interactions (Makinodan et al. 2012; Fang et al. 2022), learning, and memory (Steadman et al. 2020; Shimizu et al. 2023; Pan et al. 2020; Fang et al. 2022; Gibson et al. 2014; McKenzie et al. 2014; Benamer et al. 2020). Understanding how glia interact and regulate neuronal function during typical brain development is essential to reveal changes in neurodevelopmental disorders such as FXS. Our work is the first to report conserved and cell‐autonomous defects in both rat and FXS hPSC‐derived oligodendrocytes, providing new insights into the developmental role of FMRP in human and rat oligodendrocyte maturation while offering a first glimpse into the early oligodendrocyte dysfunction in FXS.

## Author Contributions

V.R.: Concept, Investigation, Data analysis, Writing original draft, Writing – review and editing. E.T.: Investigation, Data Analysis, Writing – review and editing. I.K.: Investigation, Data Analysis, Writing – review and editing. Z.K.: Investigation and Data analysis, Writing – review and editing. K.B.: Investigation, Writing – review and editing. B.V.: Investigation, Writing – review and editing. D.H.: Investigation, Writing – review and editing. D.S.: Investigation, Writing – review and editing. BKR: Investigation, Writing – review and editing. R.P.: Investigation, Writing – review and editing. O.D.: Concept, Investigation, Data Analysis, Writing – review and editing. P.C.K.: Concept, Writing – review and editing. SChattarji: Concept, Funding, Supervision, Writing – review and editing. B.T.S.: Supervision, Writing – review and editing. SChandran: Concept, Funding, Supervision, Writing – review and editing. L.Z.: Concept, Investigation, Data Analysis, Funding, Supervision, Writing – original draft, Writing – review and editing.

## Conflicts of Interest

The authors declare no conflicts of interest.

## Supporting information


**Figure S1.** FMRP targets in rodent oligodendrocyte lineage cell classes. Graph showing the top 25 enriched GO terms from in silico analysis of mouse oligodendrocyte‐specific datasets and FMRP gene targets in the postnatal mouse brain. GO terms were ordered by their maximum KS‐test value across all cell types. Each cell type is marked with a different cell color. The point size indicates the number of genes (i.e., FMRP mRNA targets) annotated with the GO term. Solid points indicate that the KS‐test was significant at *p* < 0.05. Oligodendrocyte clusters according to Marques et al. (2016).
**Figure S2.** Unaffected OPC proliferation and differentiation in *Fmr1* ^+/*y*^ and *Fmr1* ^*−*/*y*^ rat oligodendrocyte cultures. (A, B) Representative images of *Fmr1* ^+/*y*^ and *Fmr1* ^*−*/*y*^ rat oligodendrocyte cultures after 6 days in vitro immunostained for PDGFRα (magenta), Ki67 (green) and counterstained with Hoechst (blue). (C, D) Representative images of *Fmr1* ^+/*y*^ and *Fmr1* ^*−*/*y*^ rat oligodendrocyte cultures after 6 days in vitro immunostained for O4 (magenta) and OLIG2 (green). (E) Percentage of PDGFRα+ OPCs over the total number of Hoechst cells in *Fmr1* ^+/*y*^ and *Fmr1* ^*−*/*y*^ rat oligodendrocyte cultures. (F) Percentage of proliferating PDGFRα+ Ki67+ OPCs over the total number of PDGFRα+ cells in *Fmr1* ^+/*y*^ and *Fmr1* ^*−*/*y*^ rat oligodendrocyte cultures. (G) Percentage of differentiated O4+ oligodendrocytes over the total number of OLIG2+ cells in *Fmr1* ^+/*y*^ and *Fmr1* ^*−*/*y*^ rat oligodendrocyte cultures. Each data point is a different experiment. Error bars indicate sem; *p* values calculated using two‐tailed unpaired *t*‐test with Welch’s correction.
**Figure S3.** Gene expression analysis in *Fmr1* ^+/*y*^ and *Fmr1* ^*−*/*y*^ rat and *FMR1* ^+/*y*^ and *FMR1* ^−/*y*^ human oligodendrocyte cultures. (A) Relative expression of selected genes in day 6 *Fmr1* ^+/*y*^ and *Fmr1* ^*−*/*y*^ rat oligodendrocyte cultures. (B) Representative FACS plots showing the gating strategy for isolating O4‐positive human oligodendrocytes in 7‐day‐old cultures. C. Relative expression of selected genes in day 7 *FMR1* ^+/*y*^ and *FMR1* ^*−*/*y*^ human oligodendrocytes. Each data point is a different experiment. Error bars indicate sem; *p* value for *PAK1* and *Tppp* was calculated with two‐tailed unpaired *t*‐test.
**Figure S4.** hOPC numbers and proliferation unchanged in *FMR1* ^+/*y*^ and *FMR1* ^*−*/*y*^ glial spheres. (A) Schematic showing migration of cells from the human glial spheres. Migrating cells were enriched with OPCs and analyzed in B‐O panels. (B, C, E, F, H, I, K, L, N, O) Representative images of *FMR1* ^+/*y*^ and *FMR1* ^*−*/*y*^ human oligodendrocyte precursors after 2 days in vitro immunostained for OLIG2, PDGFRα, SOX10 and Ki67 and counterstained with DAPI (blue). (D, G, J, M) Graph showing percent of OLIG2, PDGFRα, SOX10, and Ki67 cells over total nuclei. (P) Graph showing percent of triple positive PDGFRα+Ki67+ SOX10+ cells over total nuclei. Each data point is a different experiment. Error bars indicate sem from 3 to 4 different experiments; *p* values calculated using two‐tailed unpaired *t*‐test with Welch’s correction.
**Figure S5.** Human oligodendrocytes and oligodendrocyte lineage cells unchanged in *FMR1* ^*−*/*y*^ and mFXS glial cultures. A‐B. Representative images of *FMR1* ^+/*y*^ and *FMR1* ^*−*/*y*^ human oligodendrocytes after 7 days in vitro immunostained for MBP (green), SOX10 (white) and counterstained with DAPI (blue). C‐F. Representative images of *FMR1* ^+/*y*^, *FMR1* ^*−*/*y*^, IsoFXS and mFXS Human oligodendrocytes after 7 days in vitro immunostained for SOX10 (white) and counterstained with DAPI (blue). G. Graph showing percent of human oligodendrocytes (MBP+ SOX10+ over the total SOX10+ cells). H. Graph showing percent of human oligodendrocyte lineage cells (SOX10+ over the total nuclei). Each data point is a different experiment. Error bars indicate sem from 3 different experiments; P values calculated using two‐tailed unpaired t‐test with Welch’s correction.
**Figure S6.** Oligodendrocyte cell densities are unaffected in *Fmr1* ^*−*/*y*^ rats in vivo. (A) Schematic of the ages and brain areas tested in *Fmr1* ^+/*y*^ and *Fmr1* ^*−*/*y*^ rats. (B) Ratio of PDGFRα+/OLIG2+ cells over the total OLIG2+ cells in layers 2/3 of the prefrontal cortex of *Fmr1* ^+/*y*^ and *Fmr1* ^*−*/*y*^ rats at the third postnatal week. (C) Ratio of OLIG2‐expressing cells over the total cell number in layers 2/3 of the prefrontal cortex of *Fmr1* ^+/*y*^ and *Fmr1* ^*−*/y^ rats at the third postnatal week. (D) Ratio of CC1+/OLIG2+ cells over the total OLIG2+ cells in layers 2/3 of the prefrontal cortex of *Fmr1* ^+/*y*^ and *Fmr1* ^*−*/*y*^ rats at the third postnatal week. (E, F) Deep white matter sections from P8‐12 (2nd week) *Fmr1* ^+/*y*^ and *Fmr1* ^*−*/*y*^ rats stained for OPC marker PDGFRα (magenta) and counterstained with Hoechst (blue). (G) Density of total cells (Hoechst+/mm^3^) in cerebellar deep white matter between genotypes in the second postnatal week. (H) Ratio of PDGFRα+ cells over the total Hoechst+ cells in cerebellar deep white matter of *Fmr1* ^+/*y*^ and *Fmr1* ^*−*/*y*^ rats in the second postnatal week. (I, J) MNTB sections from adult *Fmr1* ^+/*y*^ and *Fmr1* ^*−*/*y*^ rats stained for OPC marker PDGFRα (magenta), mature oligodendrocyte marker CC1 (green) and counterstained with Hoechst (blue). (K) Ratio of PDGFRα+ cells over the total Hoechst+ cells in the MNTB of *Fmr1* ^+/*y*^ and *Fmr1* ^*−*/*y*^ adult rats. (L) Ratio of CC1+ cells over the total Hoechst+ cells in the MNTB of *Fmr1* ^+/*y*^ and *Fmr1* ^*−*/*y*^ adult rats. Data presented as mean ± sem and each circle is a rat. *p* values calculated with two‐tailed, unpaired *t*‐tests with Welch’s correction.
**Figure S7.** Western blot analysis of myelin proteins in the frontal cortex of *Fmr1* ^+/*y*^ and *Fmr1* ^*−*/*y*^ rats in vivo. (A) Western blot of P21 *Fmr1* ^+/*y*^ and *Fmr1* ^*−*/*y*^ rat cortices for CNPase and MBP (4 isoforms). GAPDH as loading control and total protein are also shown. (B) Band intensity analysis for CNPase over GAPDH (left) and MBP over GAPDH (right) showed no significant changes between genotypes. Data presented as mean ± sem and each circle is a rat. *p* values calculated with two‐tailed, unpaired *t*‐tests.
**Figure S8.** Transplanted human oligodendrocytes express myelin proteins CNPase and PLP. (A–D) Representative images of transplanted *FMR1* ^+/*y*^ and *FMR1* ^*−*/*y*^ human oligodendrocytes in *MBP* ^*shi*/*shi*^, *Rag1* ^*−*/*−*^ mice at 12 weeks immunostained for MBP (green), CNPase/PLP (magenta) and counterstained with DAPI (blue). Yellow asterisks indicate myelin sheaths co‐labeled for both MBP and CNPase/PLP.

**Table S1.** Summary of all hPSC lines used in this study.

**Table S2.** Gene ontology (GO) enrichment analysis in the biological process (BP) category was performed on 2285 FMRP targets (identified in Maurin et al.) against a background universe of 7066 genes both expressed in single‐cell sequencing of oligodendrocyte lineage cells (Marques et al.) and included in the study of Maurin et al. Only GO terms with at least ten FMRP target genes were retained.

## Data Availability

All data are included in the manuscript and in the Supporting Information. The data that support the findings of this study are available from the corresponding author upon reasonable request.
