# Supplementary material for: The Fragile X Messenger Ribonucleoprotein 1 Regulates the Morphology and Maturation of Human and Rat Oligodendrocytes
Source: Glia. 2025 Feb 10;73(6):1203–20. doi: 10.1002/glia.24680 (PMC12012330; doi:10.1002/glia.24680)
Supplement: Supplementary file 1 — Figure S1. FMRP targets in rodent oligodendrocyte lineage cell classes. Graph showing the top 25 enriched GO terms from in silico analysis of mouse oligodendrocyte‐specific datasets and FMRP gene targets in the postnatal mouse brain. GO terms were ordered by their maximum KS‐test value across all cell types. Each cell type is marked with a different cell color. The point size indicates the number of genes (i.e., FMRP mRNA targets) annotated with the GO term. Solid points indicate that the KS‐test was significant at p < 0.05. Oligodendrocyte clusters according to Marques et al. (2016). Figure S2. Unaffected OPC proliferation and differentiation in Fmr1 +/y and Fmr1 −/y rat oligodendrocyte cultures. (A, B) Representative images of Fmr1 +/y and Fmr1 −/y rat oligodendrocyte cultures after 6 days in vitro immunostained for PDGFRα (magenta), Ki67 (green) and counterstained with Hoechst (blue). (C, D) Representative images of Fmr1 +/y and Fmr1 −/y rat oligodendrocyte cultures after 6 days in vitro immunostained for O4 (magenta) and OLIG2 (green). (E) Percentage of PDGFRα+ OPCs over the total number of Hoechst cells in Fmr1 +/y and Fmr1 −/y rat oligodendrocyte cultures. (F) Percentage of proliferating PDGFRα+ Ki67+ OPCs over the total number of PDGFRα+ cells in Fmr1 +/y and Fmr1 −/y rat oligodendrocyte cultures. (G) Percentage of differentiated O4+ oligodendrocytes over the total number of OLIG2+ cells in Fmr1 +/y and Fmr1 −/y rat oligodendrocyte cultures. Each data point is a different experiment. Error bars indicate sem; p values calculated using two‐tailed unpaired t‐test with Welch’s correction. Figure S3. Gene expression analysis in Fmr1 +/y and Fmr1 −/y rat and FMR1 +/y and FMR1 −/y human oligodendrocyte cultures. (A) Relative expression of selected genes in day 6 Fmr1 +/y and Fmr1 −/y rat oligodendrocyte cultures. (B) Representative FACS plots showing the gating strategy for isolating O4‐positive human oligodendrocytes in 7‐day‐old cultures. C. Relative expression of [file GLIA-73-1203-s001.docx]

**The Fragile X Messenger Ribonucleoprotein 1 regulates the morphology and maturation of human and rat oligodendrocytes**

Vidya Ramesh^1-5,11^, Eleni Tsoukala^5,6^, Ioanna Kougianou^5,6^, Zrinko Kozic^5,6^, Karen Burr^1-5^, Biju Viswanath^7^, David Hampton^1-4^, David Story^1-5^, Bharath Kumar Reddy^8^, Rakhi Pal^8^, Owen Dando^2,5,6^, Peter C Kind^5,6,9,11^, Sumantra Chattarji^5,8,10,11^, Bhuvaneish T Selvaraj^1-4^, Siddharthan Chandran^1-5,9,11^* and Lida Zoupi^5,6^*

**Supplementary Material**


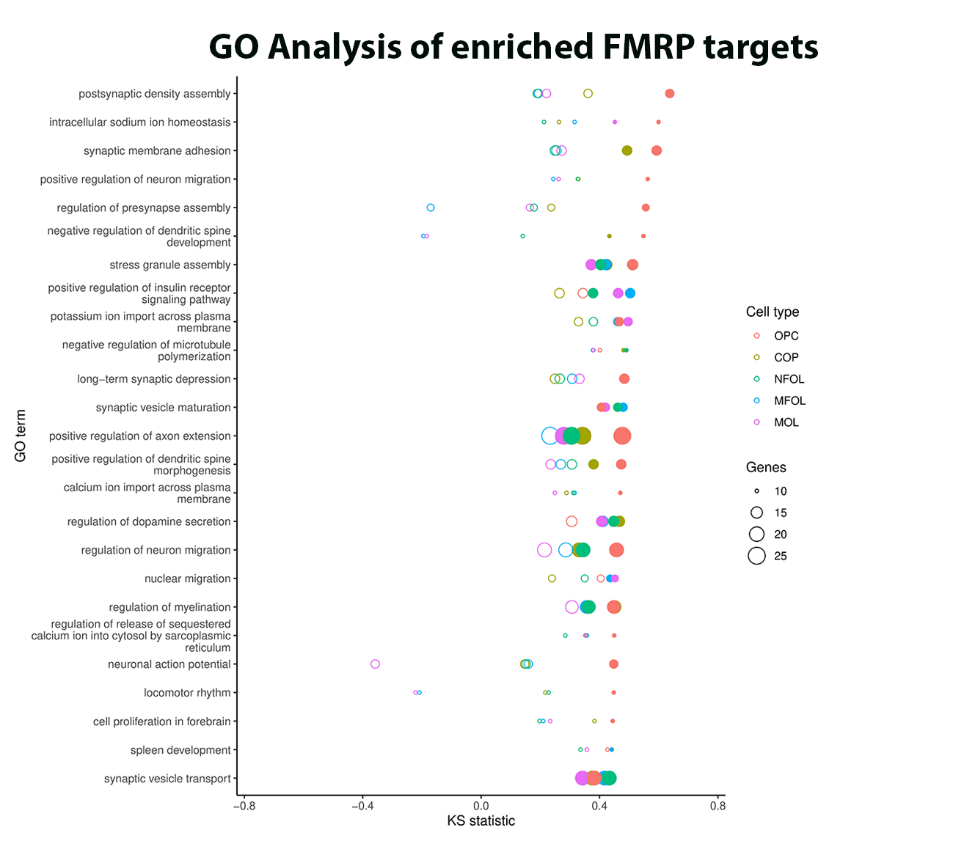


**Supplementary figure 1: FMRP targets in rodent oligodendrocyte lineage cell classes.** Graph showing the top 25 enriched GO terms from *in silico* analysis of mouse oligodendrocyte-specific datasets and FMRP gene targets in the postnatal mouse brain. GO terms were ordered by their maximum KS-test value across all cell types. Each cell type is marked with a different cell colour. The point size indicates the number of genes (i.e., FMRP mRNA targets) annotated with the GO term. Solid points indicate that the KS-test was significant at p<0.05. Oligodendrocyte clusters according
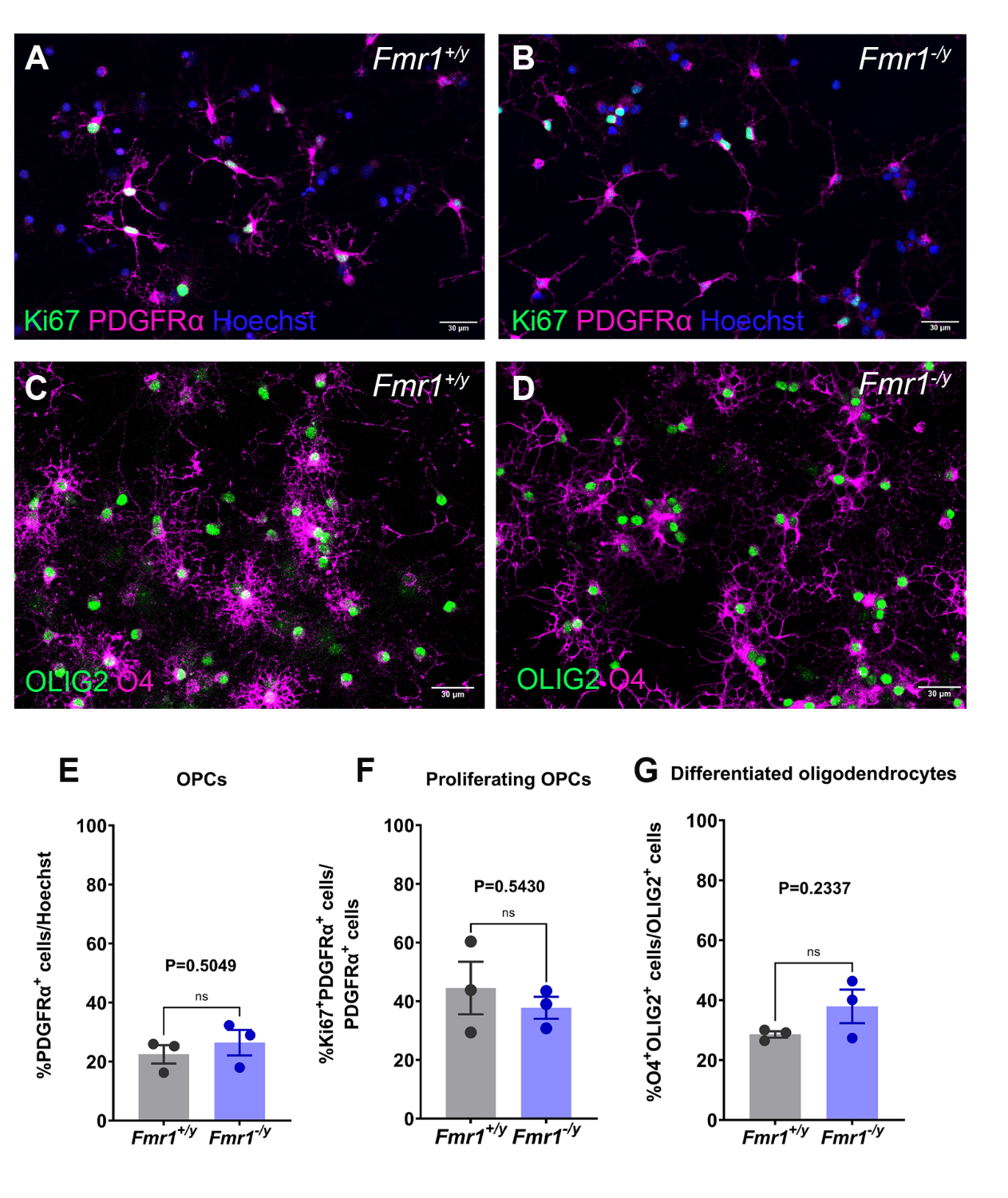
to Marques et al 2016.

**Supplementary figure 2: Unaffected OPC proliferation and differentiation in *Fmr1^+/y^* and *Fmr1^-/y^* rat oligodendrocyte cultures. A-B.** Representative images of *Fmr1^+/y^* and *Fmr1^-/y^* rat oligodendrocyte cultures after 6 days *in vitro* immunostained for PDGFRα (magenta), Ki67 (green) and counterstained with Hoechst (blue). **C-D.** Representative images of *Fmr1^+/y^* and *Fmr1^-/y^* rat oligodendrocyte cultures after 6 days *in vitro* immunostained for O4 (magenta) and OLIG2 (green). **E.** Percentage of PDGFRα+ OPCs over the total number of Hoechst cells in *Fmr1^+/y^* and *Fmr1^-/y^* rat oligodendrocyte cultures. **F.** Percentage of proliferating PDGFRα+ Ki67+ OPCs over the total number of PDGFRα+ cells in *Fmr1^+/y^* and *Fmr1^-/y^* rat oligodendrocyte cultures. **G.** Percentage of differentiated O4+ oligodendrocytes over the total number of OLIG2+ cells in *Fmr1^+/y^* and *Fmr1^-/y^* rat oligodendrocyte cultures. Each data point is a different experiment. Error bars indicate sem; P values calculated using two-tailed unpaired t-test with Welch’s correction.


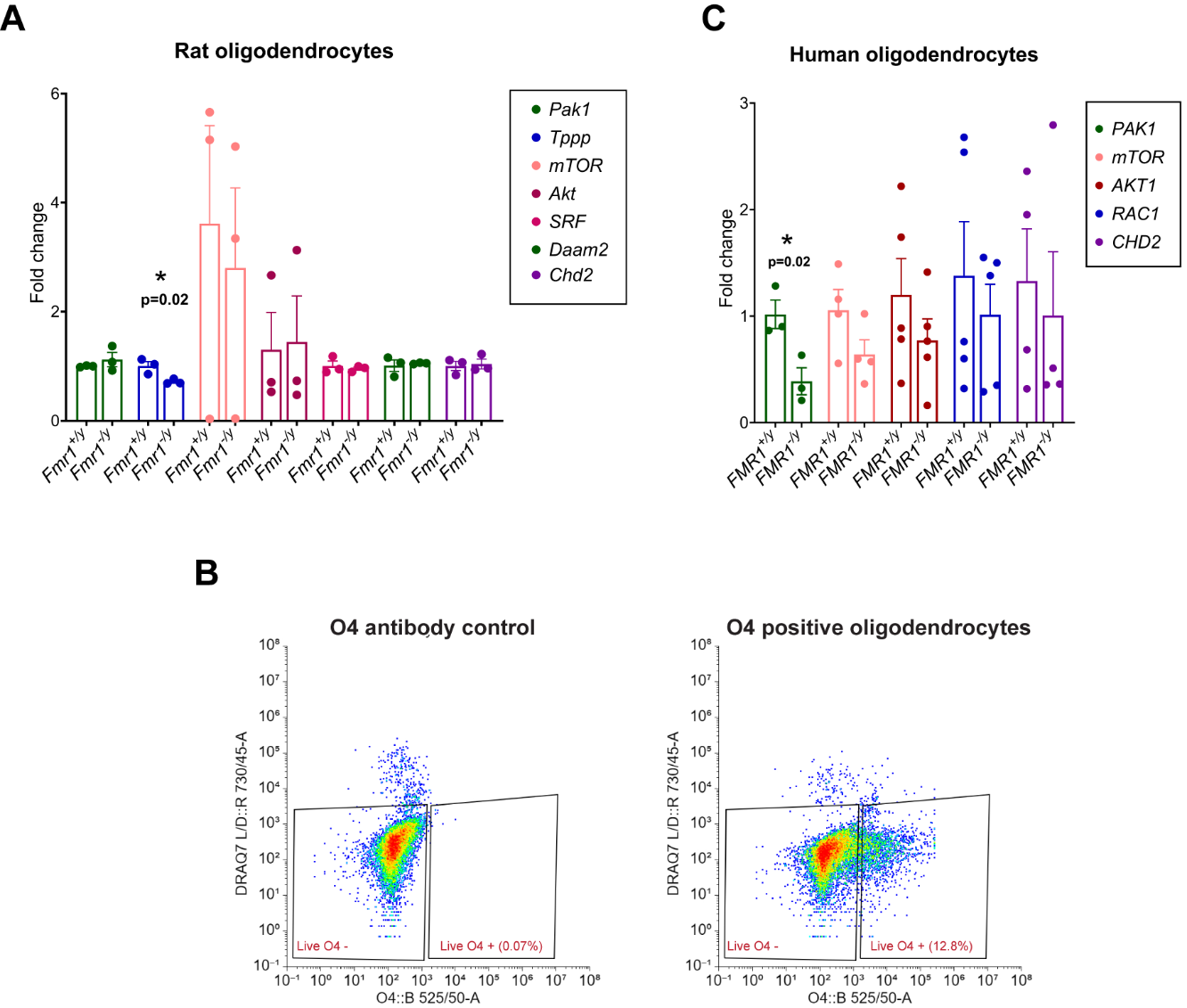


**Supplementary figure 3: *Gene expression analysis in Fmr1^+/y^ and Fmr1^-/y^ rat and FMR1^+/y^ and FMR1^-/y^ human oligodendrocyte cultures.* A.** Relative expression of selected genes in day 6 *Fmr1^+/y^* and *Fmr1^-/y^* rat oligodendrocyte cultures**. B.** Representative FACS plots showing the gating strategy for isolating O4-positive human oligodendrocytes in 7-day old cultures. **C.** Relative expression of selected genes in day 7 *FMR1^+/y^* and *FMR1^-/y^* human oligodendrocytes. Each data point is a different experiment. Error bars indicate sem; P value for *PAK1* and *Tppp* was calculated with two-tailed unpaired t-test.


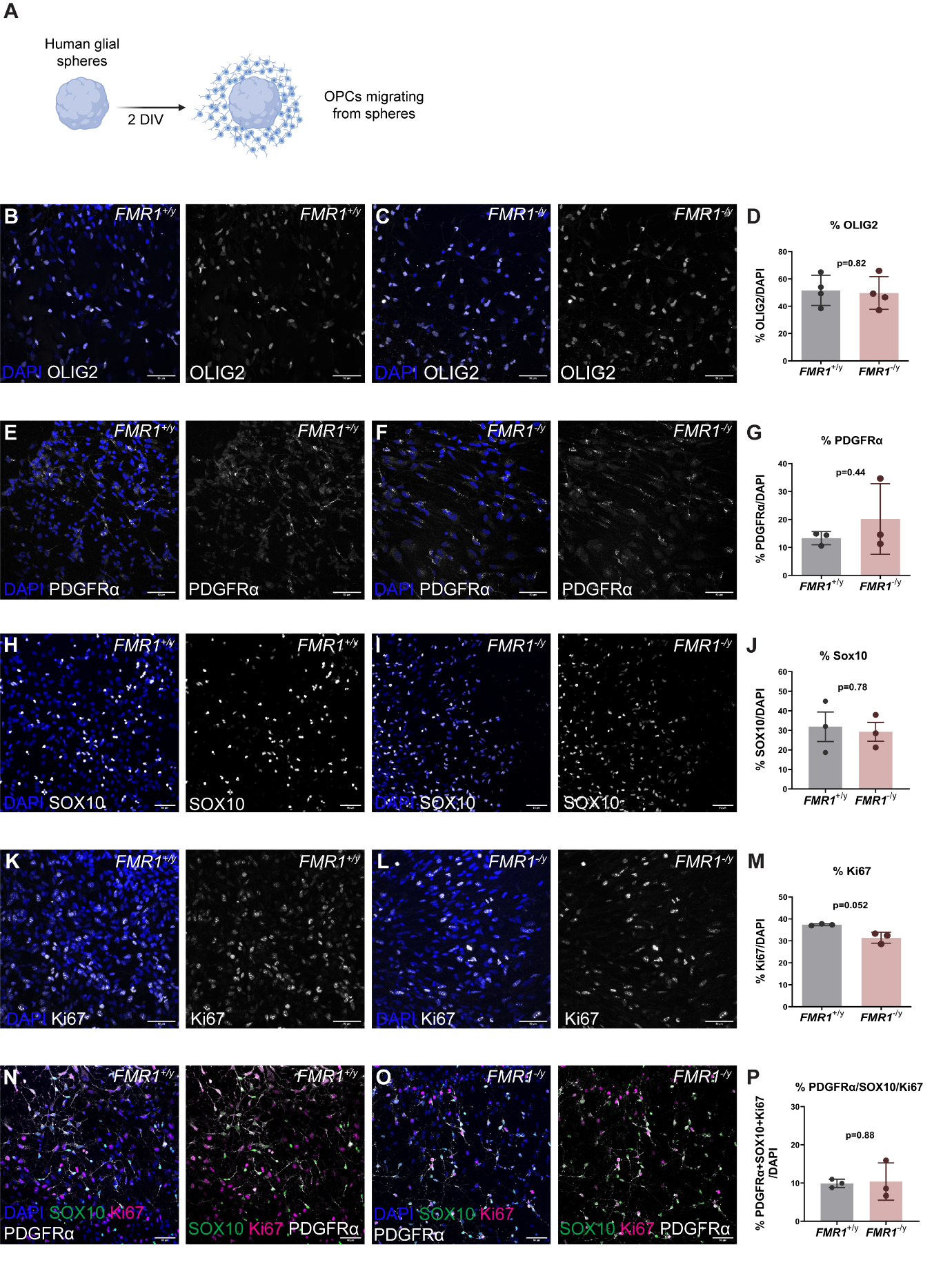


**Supplementary figure 4: hOPC numbers and proliferation unchanged in *FMR1^+/y^* and *FMR1^-/y^* glial spheres. A.** Schematic showing migration of cells from the human glial spheres. Migrating cells were enriched with OPCs and analysed in B-O panels. **B,C,E,F,H,I,K,L,N,O** Representative images of *FMR1^+/y^* and *FMR1^-/y^* Human oligodendrocyte precursors after 2 days *in vitro* immunostained for OLIG2, PDGFRα, SOX10 and Ki67 and counterstained with DAPI (blue). **D,G,J,M** Graph showing percent of OLIG2, PDGFRα, SOX10 and Ki67 cells over total nuclei. **P**. Graph showing percent of triple positive PDGFRα+Ki67+SOX10+ cells over total nuclei. Each data point is a different experiment. Error bars indicate sem from 3-4 different experiments; P values calculated using two-tailed unpaired t-test with Welch’s correction.

***
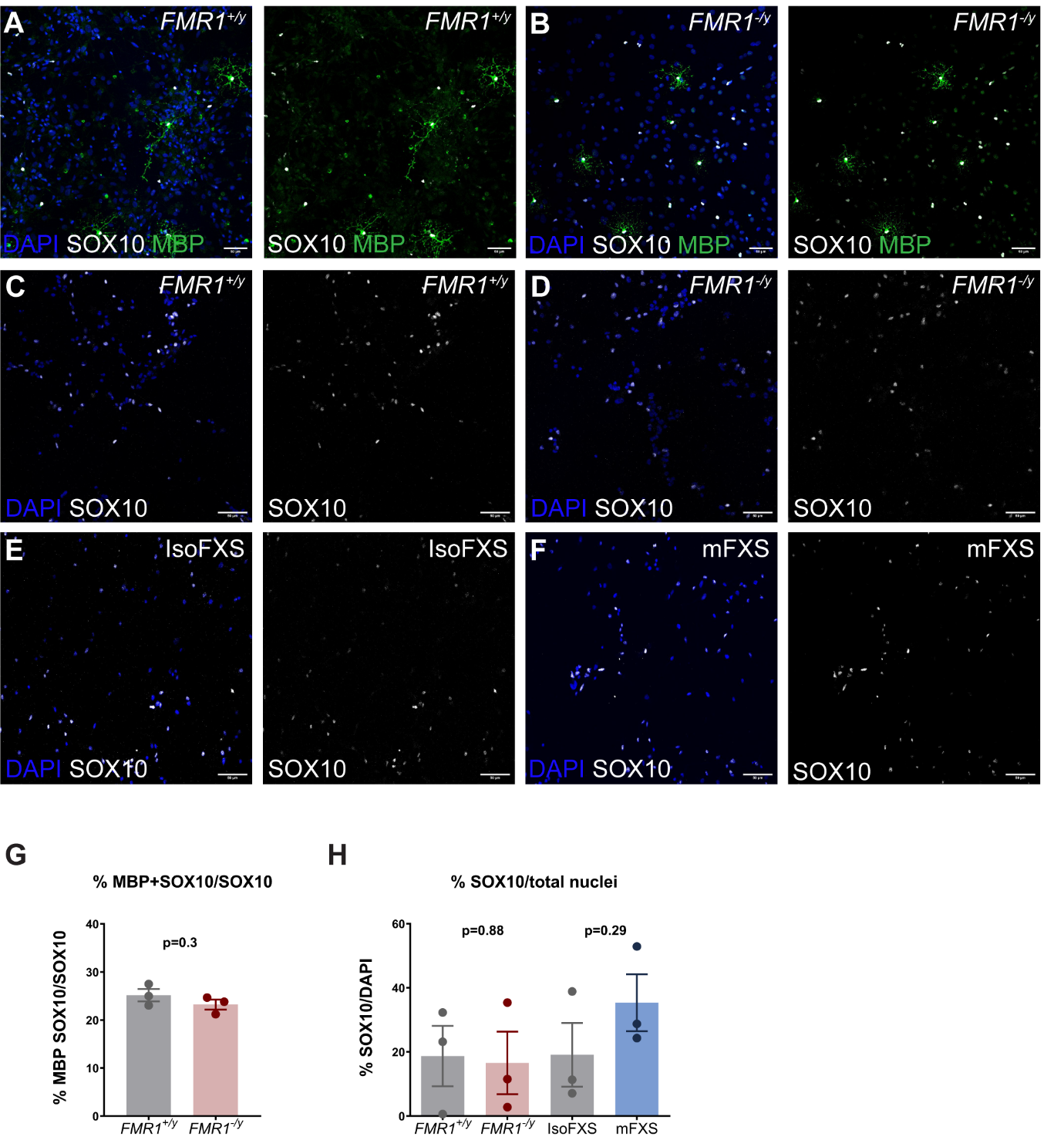
***

**Supplementary figure 5: Human oligodendrocytes and oligodendrocyte lineage cells unchanged in *FMR1^-/y^* and *mFXS* glial cultures*.* A-B.** Representative images of *FMR1^+/y^* and *FMR1^-/y^* human oligodendrocytes after 7 days *in vitro* immunostained for MBP (green), SOX10 (white) and counterstained with DAPI(blue). **C-F.** Representative images of *FMR1^+/y^, FMR1^-/y^,* IsoFXS and mFXS Human oligodendrocytes after 7 days *in vitro* immunostained for SOX10 (white) and counterstained with DAPI (blue). **G.** Graph showing percent of human oligodendrocytes (MBP+SOX10+ over the total SOX10+ cells). **H.** Graph showing percent of human oligodendrocyte lineage cells (SOX10+ over the total nuclei). Each data point is a different experiment. Error bars indicate sem from 3 different experiments; P values calculated using two-tailed unpaired t-test with Welch’s correction.


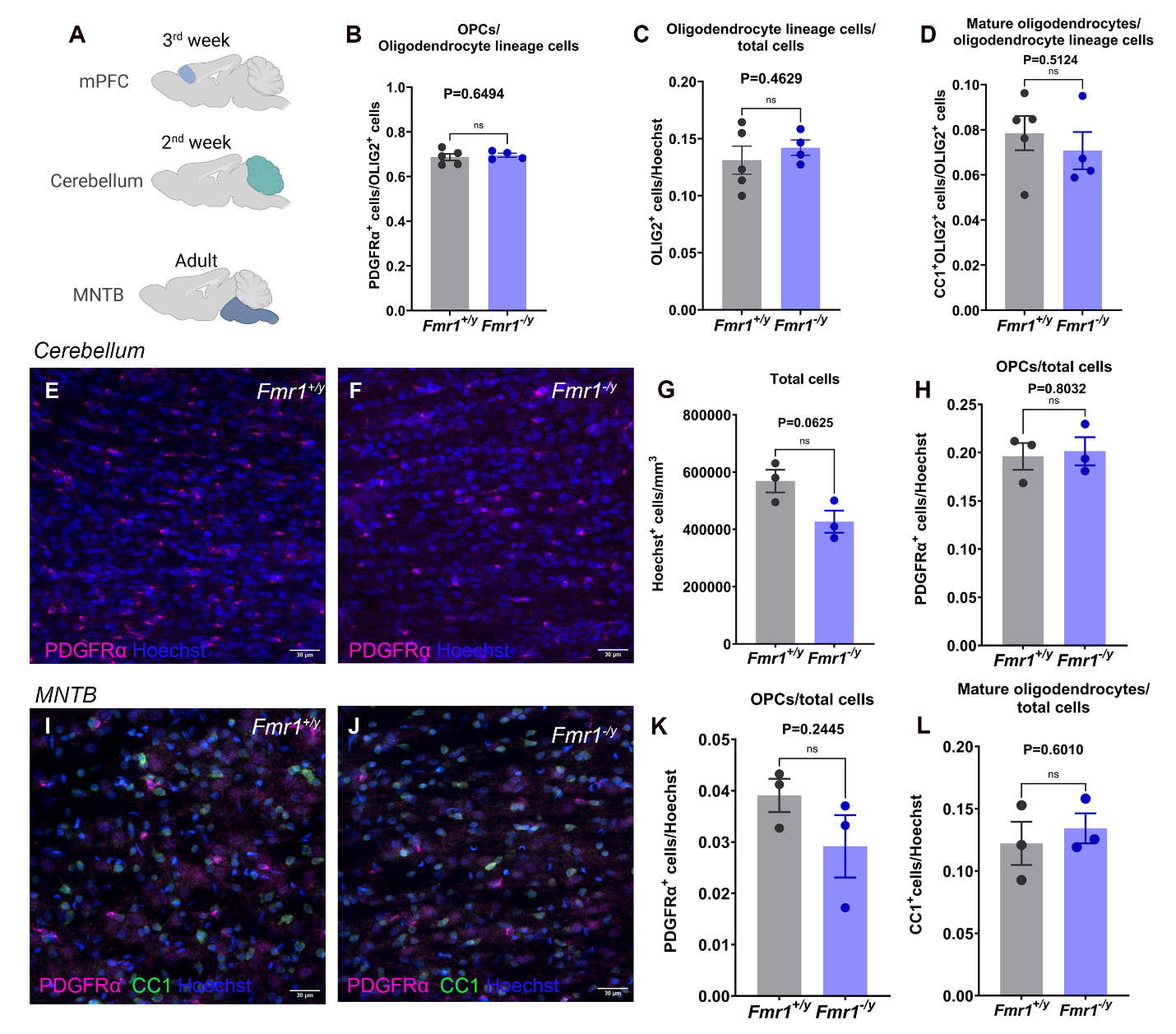


**Supplementary Figure 6: Oligodendrocyte cell densities are unaffected in *Fmr1^-/y^* rats *in vivo*. A.**  Schematic of the ages and brain areas tested in *Fmr1^+/y^* and *Fmr1^-/y^* rats. **B.** Ratio of PDGFRα+/OLIG2+ cells over the total OLIG2+ cells in layers 2/3 of the prefrontal cortex of *Fmr1^+/y^* and *Fmr1^-/y^* rats at the third postnatal week **C.** Ratio of OLIG2-expressing cells over the total cell number in layers 2/3 of the prefrontal cortex of *Fmr1^+/y^* and *Fmr1^-/^*^y^ rats at the third postnatal week. **D.** Ratio of CC-1+/OLIG2+ cells over the total OLIG2+ cells in layers 2/3 of the prefrontal cortex of *Fmr1^+/y^* and *Fmr1^-/y^* rats at the third postnatal week. **E-F.** Deep white matter sections from P8-12 (2^nd^ week) *Fmr1^+/y^* and *Fmr1^-/y^* rats stained for OPC marker PDGFRα (magenta) and counterstained with Hoechst (blue). **G**. Density of total cells (Hoechst+/mm^3^) in cerebellar deep white matter between genotypes in the second postnatal week. **H.** Ratio of PDGFRα+ cells over the total Hoechst+ cells in cerebellar deep white matter of *Fmr1^+/y^* and *Fmr1^-/y^* rats in the second postnatal week. **I-J.** MNTB sections from adult *Fmr1^+/y^* and *Fmr1^-/y^* rats stained for OPC marker PDGFRα (magenta), mature oligodendrocyte marker CC1 (green) and counterstained with Hoechst (blue). **K**. Ratio of PDGFRα+ cells over the total Hoechst+ cells in the MNTB of *Fmr1^+/y^* and *Fmr1^-/y^* adult rats. **L**. Ratio of CC1+ cells over the total Hoechst+ cells in the MNTB of *Fmr1^+/y^* and *Fmr1^-/y^* adult rats. Data presented as mean±sem and each circle is a rat. P values calculated with two-tailed, unpaired t-tests with Welch’s correction.

**Supplementary Figure 7: Western blot analysis of myelin proteins in the frontal cortex of *Fmr1^+/y^* and *Fmr1^-/y^* rats *in vivo*. A.**  Western blot of P21 *Fmr1^+/y^* and *Fmr1^-/y^* rat cortices for CNPase and MBP (4 isoforms). GAPDH as loading control and total protein are also shown. **B.** Band intensity analysis for CNPase over GAPDH (left) and MBP over GAPDH (right)
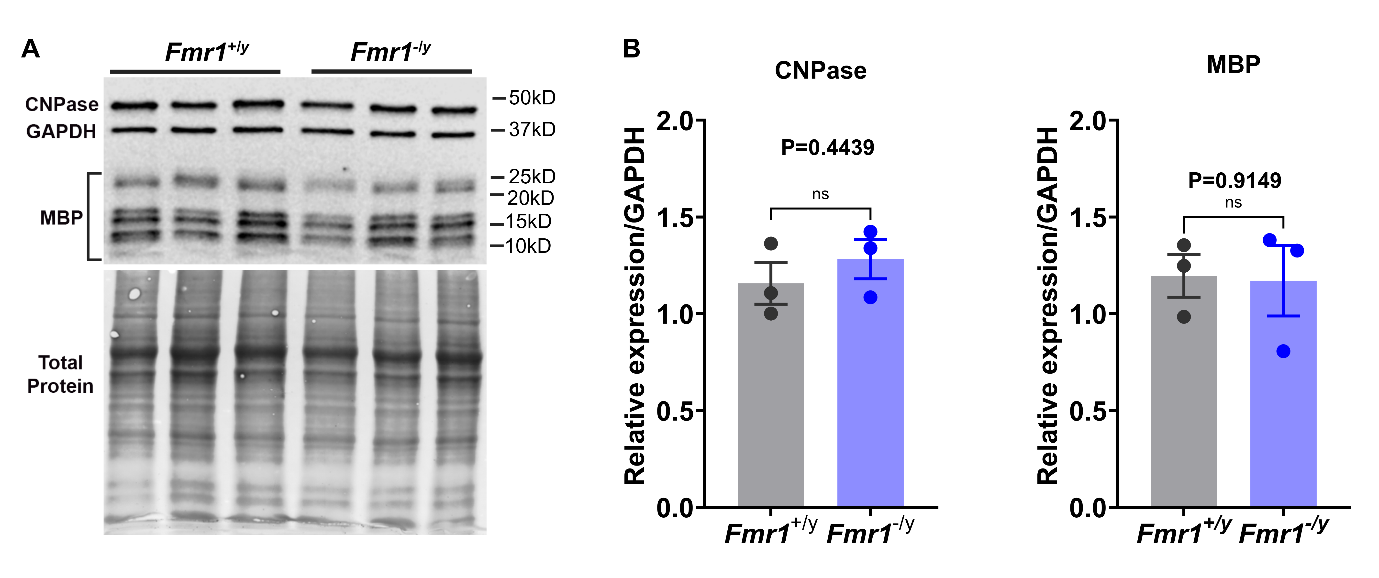
showed no significant changes between genotypes. Data presented as mean±sem and each circle is a rat. P values calculated with two-tailed, unpaired t-tests.


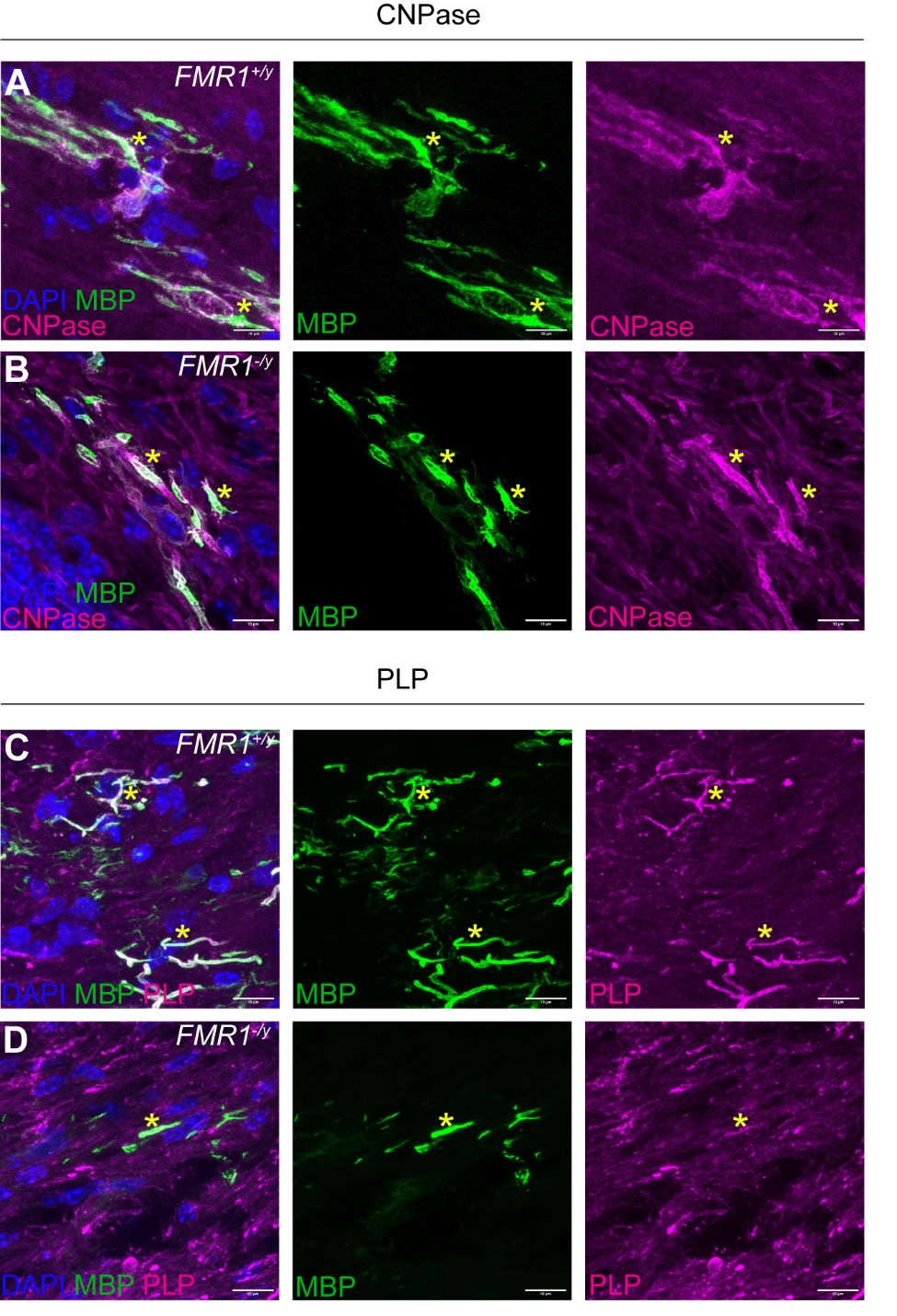


**Supplementary figure 8: Transplanted human oligodendrocytes express myelin proteins CNPase and PLP. A-D** Representative images of transplanted *FMR1^+/y^* and *FMR1^-/y^* human oligodendrocytes in *MBP^shi/shi^*,*Rag1^-/-^* mice at 12 weeks immunostained for MBP (green), CNPase/PLP (magenta) and counterstained with DAPI (blue). Yellow asterisks indicate myelin sheaths co-labelled for both MBP and CNPase/PLP.
