# Supplementary material for: The Fragile X Messenger Ribonucleoprotein 1 Regulates the Morphology and Maturation of Human and Rat Oligodendrocytes
Source: Glia. 2025 Feb 10;73(6):1203–20. doi: 10.1002/glia.24680 (PMC12012330; doi:10.1002/glia.24680)
Supplement: Supplementary file 2 — Table S1. Summary of all hPSC lines used in this study. [file GLIA-73-1203-s002.docx]

| Name on manuscript | Source | Identifier |
| --- | --- | --- |
| *FMR1^+/y^* | UK Stem Cell Bank | Shef4 |
| *FMR1^-/y^* | Parental line: *FMR1^+/y^*  Gene-edited: University of Edinburgh | Shef4-FMR1 null |
| mFXS | Fibroblasts; Coriell Institute for Medical Research (GM07072)  Reprogrammed: Cedars-Sinai RMI iPSC Core | CS072iFXS-n4 |
| IsoFXS | Parental line: mFXS  Gene-edited: University of Edinburgh | n/a |

**Supplementary table 1 – Summary of all hPSC lines used in this study**
